# Butyl Acrylate/2‐Methylene‐1,3‐Dioxepane/Vinyl Acetate Emulsion Terpolymerization: Incorporating Backbone Degradable Linkages into Adhesive Applications

**DOI:** 10.1002/cssc.202402478

**Published:** 2025-01-08

**Authors:** Maryam Movafagh, Kelly M. Meek, Marc A. Dubé

**Affiliations:** ^1^ Department of Chemical and Biological Engineering University of Ottawa Ottawa ON Canada; ^2^ Department of Chemical and Materials Engineering Concordia University Montreal QC Canada

**Keywords:** 2-methylene-1,3-dioxepane (MDO), Radical ring-opening polymerization, Pressure-sensitive adhesive (PSA), Emulsion polymerization, Butyl acrylate, Vinyl acetate

## Abstract

The ring‐opening polymerization of bio‐based monomer 2‐methylene‐1,3‐dioxepane (MDO) can reportedly enhance polymer degradability. Butyl acrylate (BA)/MDO/vinyl acetate (VAc) terpolymers were synthesized via emulsion polymerization for their eventual application as pressure‐sensitive adhesives (PSAs). While using MDO in emulsion polymerization leads to a more sustainable process, it also presents challenges such as MDO hydrolysis, MDO ring retention, and inadequate MDO distribution. By carefully selecting reaction conditions such as pH and temperature, MDO hydrolysis and MDO ring retention were mitigated, and a uniform distribution of MDO throughout the terpolymer was confirmed. In addition, carboxylated cellulose nanocrystals (cCNCs) were incorporated into the final formulation to enhance the PSA properties.

## Graphical abstract

The radical ring‐opening emulsion polymerization of BA/MDO/VAc was investigated, and a more sustainable process for producing degradable adhesives was focused on. By controlling pH and temperature, both MDO hydrolysis and ring retention were effectively mitigated. The final product had a uniform MDO distribution in the polymer backbone and resulted in improved adhesive properties aligning with green chemistry principles.

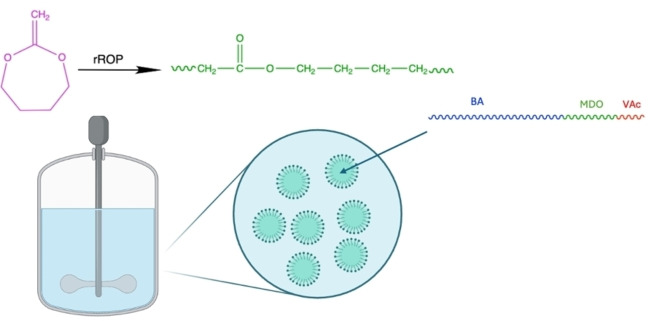

## Introduction

Polymer materials offer unrivalled benefits to humanity, meeting a variety of needs over a range of applications. However, due to their widespread use and existing production and disposal methods, polymers present substantial environmental issues by contributing to climate change, air pollution, waste generation, and water pollution.^[1]^ This motivates high interest in producing biodegradable polymers such as polyesters.[^2^, ^3^] Aliphatic polyesters are prominent in the category of biodegradable polymers because of their hydrolyzable ester bonds and comparatively short aliphatic chains within the macromolecules, making them significant examples of polymers which align with the principles of environmental sustainability.^[4]^ Ionic ring‐opening polymerization, polycondensation, and polyaddition methods are well‐known for producing such biodegradable polyesters.[^5^, ^6^] However, all these approaches have limitations, including heightened sensitivity to impurities, increased operational costs, and restricted monomer applicability, which must be carefully navigated to exploit their full potential.^[7]^

Another method of producing polyesters is via radical ring‐opening polymerization, a chain‐growth polymerization method. One example of radical ring‐opening polymerization uses cyclic ketene acetals (CKAs).[^8^, ^9^] Aliphatic polyesters, made via ring‐opening polymerization of CKAs, are the most studied biodegradable polymers. Because of the molecular structure of CKAs, the resulting polymers will have ester groups along their backbone, which should enhance biodegradation compared to polymers possessing backbones solely consisting of carbon‐carbon linkages. The disadvantage of CKAs is the conflict between the ring‐opening process and the unintended direct vinyl propagation that results in polyacetals (aka ring retention). To increase the selectivity of the ring‐opening process, two criteria were initially considered while designing a monomer for radical ring‐opening polymerization: (i) the ring strain and (ii) the stabilization of the radical when the ring is opened. For instance, it is known that 6‐membered ring monomers have challenging ring‐opening polymerizations. This is explained by the reduced ring strain of cyclohexane‐like monomers for which the ring strain is zero.^[10]^ This situation has led to the predominant use of two cyclic monomers: seven‐membered ring CKAs such as MDO and the 5,6‐benzo‐2‐methylene‐1,3‐dioxepane (BMDO). Both MDO and BMDO are known to undergo complete ring‐opening polymerization over a broad range of reaction conditions. MDO can be an excellent candidate for ring‐opening polymerization because it has (i) an increased steric hindrance of the ring‐held radical compared to the stability of the ring‐open radicals and (ii) a propensity for ring‐open radicals to undergo backbiting reactions to create more stable radicals.^[11]^ In addition, MDO is a bio‐based monomer because it is produced from starting materials such as 1,4‐butanediol and diethylene glycol, which are themselves bio‐sourced materials.^[12]^

Free radical copolymerization involving CKA with vinyl monomers has been previously established to create polymers with degradable backbones.[^9^, ^13^] In our current work, we aim to incorporate MDO monomer into the backbone of a vinyl terpolymer. Historically, achieving high incorporation rates of cyclic ketenes such as MDO in copolymerization with common vinyl monomers has been challenging, partly attributed to unfavorable reactivity ratios that limit their compatibility.^[8]^ Despite these difficulties, our recent findings demonstrate successful bulk terpolymerization of butyl acrylate (BA), MDO, and vinyl acetate (VAc), achieving substantial MDO incorporation within a BA‐rich terpolymer backbone.^[14]^

Due to their hydrolytic instability, CKAs are mainly copolymerized in non‐nucleophilic organic solvents; there are only a few reports on the polymerization of CKA in water and in emulsion polymerization.[^15^, ^16^] To align with green chemistry principles,^[17]^ which advocate for the minimization of solvent use, there is a significant interest in exploring solvent‐free methods such as emulsion polymerization. This technique is distinguished for its ability to produce aqueous resins with diverse colloidal and physicochemical properties. Monomer, water, surfactant (i. e., emulsifier), and a water‐soluble initiator make up a classic emulsion polymerization formulation. This heterogeneous free radical polymerization process begins with an oil‐in‐water emulsifier stabilizing the comparatively hydrophobic monomer in water, followed by the initiation reaction, ultimately leading to the nucleation of polymer particles. Water, as the continuous phase, is vital in emulsion polymerization because it keeps the viscosity low, acts as an efficient heat sink, and serves as a conduit for the transfer of monomers. Common surfactants are long‐chain hydrocarbons with hydrophobic tails and hydrophilic heads and serve to keep the polymer particle dispersion stable. Different surfactant types, such as ionic (anionic and cationic) and non‐ionic (typically polymeric), may be utilized. Water‐soluble initiators, such as potassium persulfate (KPS) and redox systems (for low‐temperature polymerization), are extensively employed.[^18^, ^19^]

Carter et al. presented a method for making waterborne and biodegradable latex polymers using MDO.^[20]^ Because of the presence of backbone ester groups, emulsion polymerization of vinyl acetate (VAc) with MDO yielded hydrolytically degradable polymer particles and latex‐based coatings. They found that a successful emulsion copolymerization of MDO could be accomplished by (i) suppressing MDO hydrolysis in the water phase by controlling pH and temperature, and (ii) establishing a process that runs at high instantaneous monomer conversions to promote MDO incorporation.^[20]^ To avoid MDO hydrolysis, it is important to run the polymerization at a low temperature (e. g., 40 °C) and under mildly basic conditions (e. g., pH=8). On the other hand, under alkaline conditions, VAc monomer can hydrolyze. They also used a redox initiation system to allow for a reduced polymerization temperature and reaction time. This new class of latex was well‐suited for developing next‐generation biodegradable and compostable single‐use food service items and other applications requiring the degradation of polymer‐based films and coatings.^[20]^

Kordes et al., in a study on the copolymerization of MDO and VAc, cited the challenges of producing MDO‐based polymers in both batch and semi‐batch emulsion polymerization.^[21]^ However, there is evidence that semi‐batch emulsion polymerization can result in reduced MDO hydrolysis by minimizing MDO‐water contact.[^20^, ^22^, ^23^] Beyond controlling MDO hydrolysis, achieving a uniform distribution of MDO within the polymer backbone is crucial for producing a degradable polymer. In our terpolymer system, which comprises BA, VAc, and MDO, each monomer pair exhibits unique reactivity ratios and distinct tendencies for homo‐propagation versus cross‐propagation with the other monomers. In a previous study of the terpolymer reactivity ratios for this system, we demonstrated that batch polymerization was indeed sufficient to generate polymer chains with significant and uniform levels of MDO.^[14]^ This has opened the door to the use of batch polymerization while controlling MDO hydrolysis and achieving significant MDO incorporation into the BA/MDO/VAc terpolymer.[^11^, ^24^]

One class of polymeric materials of interest in this research is pressure sensitive adhesives (PSAs). PSAs can quickly adhere to solid surfaces under low pressure (i. e., finger pressure) and are viscoelastic polymeric materials with a low glass transition temperature (*T_g_*) ranging from −70–−20 °C.^[25]^ They are used for labels, tapes, films, and other specialized applications, making them vital in everyday life.^[26]^ The adhesive and cohesive properties of PSAs are closely tied to their viscoelastic properties.^[27]^ Firstly, the wetting of the substrate and the Van der Waals and polar forces between the PSA and the substrate give rise to adhesion. On the other hand, intermolecular forces, and covalent interactions, such as polymer crosslinks, influence the cohesion of a PSA. Often, these forces are impacted by various production factors in opposing ways, making the control of their properties challenging. To adjust adhesive characteristics in PSA formulations, a “hard monomer” (i. e., high polymer *T_g_*), such as methyl methacrylate (MMA), can be copolymerized with a “softer” monomer (i. e., low polymer *T_g_)* such as 2‐ethyl hexyl acrylate (EHA).[^28^, ^29^] The worldwide demand for PSAs continues to grow.^[30]^ The PSA market is dominated by polyacrylates made by free radical polymerization. Acrylic adhesives have the benefits of transparency, outstanding cohesive strength, adequate tack and peel strength, and good heat and aging resistance. The “soft” monomers typically provide the required flowability and wettability to give the PSA film adequate tack and peel strength. The PSA gains hardness and internal strength from the “hard” monomers.^[31]^

Historically, PSAs have been produced using solution polymerization, which presents evident hazards both at the synthesis stage and during product application. As mentioned, increased environmental concerns on the management of volatile organic compounds (VOCs) have resulted in tremendous growth in the use of emulsion polymerization to make latexes for water‐borne adhesive applications as alternatives to solvent‐borne adhesives.^[32]^ In previous work, we successfully developed PSAs containing BA and/or VAc via emulsion polymerization. Because of our desire to make compostable PSAs, the use of MDO in the formulation makes sense. Not only would MDO enhance compostability, as discussed above, but its low homopolymer *T_g_* (−59 °C),^[33]^ makes it an ideal replacement for petroleum‐based monomers in PSA formulations. Moreover, the potential for MDO to be used in an emulsion polymerization allows us to align with several important sustainability principles. Thus, we propose to displace some of the petroleum‐based, non‐biodegradable components in a PSA formulation with MDO.^[34]^

In this work, we demonstrate the synthesis, characterization, and application of BA/MDO/VAc emulsion terpolymers, focusing on the innovative use of MDO in emulsion polymerization to develop next‐generation, environmentally friendly PSAs.

## Materials and Methods

### Materials

BA (≥99 %) and VAc (≥99 %) were purchased from Sigma‐Aldrich (Oakville ON, Canada). BA and VAc monomers were passed through inhibitor removal columns acquired from Sigma‐Aldrich. This process effectively eliminated any traces of inhibitors, such as hydroquinone or monomethyl ether hydroquinone. MDO was obtained from Wacker Chemie (München, Germany). TERGITOL^TM^ 15‐S40 surfactant (70 wt.% in water), 2‐acrylamido‐2‐methyl‐1‐propanesulfonic acid sodium salt solution (Na‐AMPS, 50 wt.% in water), ammonium persulfate (APS, 98 %), tert‐butyl hydroperoxide (t‐BHP, 70 wt.% in water), iron (II) sulfate heptahydrate (≥99 %), ethylenediaminetetraacetic acid (EDTA, >98.5 %), tetrahydrofuran (THF, ≥99.9 %), deuterated chloroform (CDCl_3,_ ≥99.9 %), and ammonium hydroxide (28.0–30.0 wt.%) were obtained from Sigma‐Aldrich. Bruggolite FF6 M reducing agent was obtained from Brüggemann (Newtown Square PA, USA). DextraCel^TM^ (i. e., carboxylated CNCs or cCNCs) was obtained from Anomera Inc. (Montreal, QC) in a “never‐dried” form (6.6 wt.% suspension in water). The carboxylate groups on the cCNCs’ surface were in the sodium‐salt form. The “apparent” cCNC diameter via dynamic light scattering (DLS) was 102±0.4 nm, and the carboxylate group content via conductimetric titration was 141±10 mmol.^[35]^ Deuterated water (D_2_O, ≥99.96 %) was obtained from Cambridge Isotopes Labs (Tewksbury MA, USA). The wetting agent, bis(2‐ethylhexyl) sulfosuccinate sodium salt (≥95 %), was obtained from TCI America (Portland OR, USA). Distilled de‐ionized water (DDW) with a resistivity of 18.0±0.2 MΩ cm was used for synthesis and characterization purposes. All reagents and chemicals were used as received unless otherwise specified.

### Methods

#### Emulsion Terpolymerization of BA/MDO/VAc

The emulsion terpolymerization formulation (Table 1) was developed by combining ideas from our previous work with PSAs,[^36^, ^37^, ^38^] and that from Carter et al.^[20]^ PSAs were synthesized using a batch emulsion polymerization process conducted under a nitrogen atmosphere in a 1.25 L stainless steel reactor (Mettler Toledo LabMax Automatic Lab Reactor). The reactor contents were mixed at 200 rpm and maintained at a constant temperature of 40 °C. A surfactant mixture was initially prepared by combining TERGITOL^TM^ 15‐S40 (16 wt.% in DDW) and Na‐AMPS with DDW. Na‐AMPS was added to provide colloidal stability. According to Mothe et al.,^[23]^ neutral surfactants such as TERGITOL^TM^ 15‐S40 are also effective in promoting latex stability. The surfactant mixture was stirred for approximately 30 minutes. Following this, the surfactant mixture and additional DDW were added to the reactor under a nitrogen blanket at the process conditions. Subsequently, an iron (II) sulfate heptahydrate solution (0.15 wt.% in DDW) and an EDTA solution (1 wt.% in DDW) were added to the mixture at 40 °C. Due to the poor solubility of EDTA in water, a few drops of ammonium hydroxide were added to enhance its solubility. A redox system comprising solutions of t‐BHP, APS (6.3 wt.% in DDW), and Bruggolite FF6 M (8 wt.% in DDW), a sulfinic acid‐based reductant, was charged to the reactor. The pH of the reactor contents was then adjusted to between 7.8 and 9 using ammonium hydroxide and stabilized for 2 minutes at 40 °C before introducing the monomer mixture. For all runs, the BA/VAc ratio in the monomer mixture was 9 : 1 (w/w), with MDO concentrations varying from 0–20 wt.%. After 70 minutes of polymerization, an additional shot of the redox system was administered to ensure complete monomer conversion and to accelerate the polymerization process. The reactor process conditions were maintained for an additional 20 minutes at 40 °C before being cooled to room temperature. Throughout the 90 minute polymerization process, the pH was kept within a range of 7.8–8.8 by periodic adjustments with ammonium hydroxide.[^21^, ^23^] It was important to maintain the pH in this range to suppress the hydrolysis of MDO as well as the other monomers. MDO hydrolysis is significantly reduced under alkaline conditions compared to neutral or acidic environments.[^21^, ^22^, ^39^]

**Table 1:** Emulsion polymerization formulation.

Component	Amount^[a]^
**Monomer mixture**: BA/MDO/VAc	~218 g
**Surfactant mixture**: TERGITOL^TM^ 15‐S40 Na‐AMPS (surfmer)	3.50 0.64
**Initiators**: t‐BHP/APS Bruggolite FF6 M **Initiator shot**: t‐BHP/APS Bruggolite FF6 M	0.31/0.75 1.44 0.04/0.09 0.18
**Reducing agent**: iron (II) sulfate heptahydrate EDTA	0.01 0.01
**Buffer**: ammonium hydroxide^[b]^	~1
**DDW (total)** ^[c]^	379 g

^[a]^ All amounts in phm (parts per hundred parts monomer) unless otherwise indicated. ^[b]^ Ammonium hydroxide quantity varied to keep the pH between 7.8 and 8.8. ^[c]^ DDW distributed in various solutions as per details in the procedure.

A dual‐channel pH meter (Fisher Scientific/Accumet Research, model AR50) was utilized to monitor the pH of samples collected throughout the reaction process and of the final latex. Offline pH measurement was used instead of a direct probe because measurement inaccuracies could arise due to the formation of a thin latex film on the probe.^[21]^ During the reaction, samples were taken periodically to monitor pH, particle size and distribution, monomer conversion, and terpolymer composition. There was no visible coagulum in all samples and the final latex; therefore, the samples were not filtered. The final latex was further characterized for gel content, degree of ring‐opening/retaining, degree of MDO hydrolysis, *T_g_*, and PSA performance (tack, peel strength, shear adhesion). After dilution in THF, the dried polymer was unfilterable (0.4 μm filter), and molecular weight analysis was impossible.

### Characterization

#### Solids Content and Monomer Conversion

Samples were collected throughout the reaction, and a few drops of hydroquinone solution (~0.1 g of 0.8 wt.% aqueous solution) were added to quench the reaction. To calculate the solids content, about 2 g of latex was weighed in an aluminum dish and dried at ambient temperature in a fume hood until a constant weight was achieved (~3–4 days). The solids content was calculated as:

(1) Solidscontent(%)=Wdriedsample/Wlatex

The instantaneous monomer conversion was then calculated based on the amount of monomer and other ingredients charged to the reactor:

(2) $$X=\frac{Solid\;content-w_{I}-w_{surf}}{w_{m}}$$

Where ***w**_l_*, ***w**_surf_*, and ***w**_m_* represent the weight fraction of initiator, surfactant, and monomer respectively, introduced to the reaction mixture by that time in the process. Overall conversion was calculated via Equation (2) by replacing the weight fractions with the total weight fractions of the ingredients added over the course of the entire reaction.

#### MDO Hydrolysis Residue and Volatile Content

Thermogravimetric analysis (TGA) was conducted to detect MDO hydrolysis products and any remaining volatile compounds, such as unreacted monomers, which may persist after the drying process. As explained above, polymer samples were prepared at ambient temperature under the fume hood. TGA platinum pans were torched and tared using the TGA 550 (TA Instruments), and samples were subsequently placed on the prepared pans for testing.

#### Particle Size and Distribution

Dynamic light scattering (DLS) by Malvern NanoS Zetasizer 2000 at an angle of 176° was used to measure the size and size distribution (PDI) of the polymer particles. Samples were prepared by diluting a drop of latex in approximately 5 mL DDW. Each measurement was taken as an average of 3 measurements.

#### Polymer Composition

A Bruker 400 MHz ^1^H‐NMR spectrometer was used to measure the terpolymer composition. Dried polymer samples were dissolved in CDCl_3_ at a ratio of 0.02 g polymer per 1.5 g solvent. The spectrometer was configured for 1D analysis, generating 32 scans.

#### Degree of Ring‐Opening/Retaining

Quantitative ^13^C‐NMR spectroscopy experiments were performed to assess the degree of ring‐opening versus ring retention in the final dried latex samples. The experiments were performed at 600 MHz using a 90° pulse with inverse gated proton decoupling, a 60 second recycle delay while collecting 472 transients. The samples were dissolved in CDCl_3_. The ^13^C‐NMR experiment was repeated on a single sample with a 120 second recycle delay, and the results suggested that the spin system was fully relaxed after 60 seconds.

#### MDO Hydrolysis

The analysis of MDO hydrolysis was performed on final latex samples using ^1^H‐NMR spectroscopy on an Avance IIIHD 600 MHz NMR spectrometer equipped with a cryoprobe. D_2_O was used as the solvent to facilitate the suppression of proton signals from water. A modified Watergate pulse sequence with gradients was used to suppress the ^1^H signals from water using an 8 ms ^1^H 90° pulse, collecting 64 transients with a 1 second recycle delay and a 1.70 s acquisition time.[^40^, ^41^]

#### Glass Transition Temperature

Dried polymer (5–10 mg) was weighed and sealed in an aluminum pan. An empty sealed pan was used as a reference. A differential scanning calorimeter (DSC) (Model Q1000 from TA Instruments) was used to measure the polymer *T_g_*. The *T_g_* was calculated from the inflection point in the reverse heat flow curve using the software provided. DSC was performed over a temperature range of T=−100–100 °C at a heating rate of 10 °C/min under a nitrogen atmosphere. The thermal history of each sample was erased in the first cycle by cooling to −100 °C, followed by heating to 100 °C, and then cooling to −100 °C. All thermal transitions were assigned from the second heating cycle.

#### Gel Content

Approximately 0.03 g of dried polymer was weighed and heat‐sealed in a poly(vinylidene fluoride) Durapore‐coated membrane pouch. The membrane pouch was then soaked in 80 mL THF in a 100 mL capped glass vial for 14 h. The glass vial was then shaken for 24 h using a mechanical shaker. The membrane pouch was then removed and dried in a fume hood until it reached a constant weight. The remaining dry gel was weighed, and the gel content calculated as:

(3) $$Gel\;content\;\left(\%\right)=\frac{W_{dried\;gel\;and\;pouch}-W_{dry\;pouch}}{W_{dry\;polymer\;and\;pouch}-W_{dry\;pouch}}$$

#### PSA Performance

Pressure Sensitive Tape Council standards (PSTC) including PSTC16 for tack, PSTC101 for peel strength, and PSTC107 A for shear adhesion were followed for specimen preparation and testing procedures for measurement of PSA properties. Final PSA latexes were cast on 30 μm corona‐treated Mylar sheets using a Meyer rod and dried at controlled conditions (temperature: 23±1 °C, and relative humidity: 50±5 %) for 48 hours before the test (2 films per sample). To enhance the film‐casting process, a wetting agent, bis(2‐ethylhexyl) sulfosuccinate sodium salt, was added to the latex formulation at a concentration of 0.2 wt.% of the total latex. The latex mixture was stirred overnight. The final dried film thicknesses, expressed as a weight per unit area, were measured and found to be uniform, with a film weight of approximately 20 g/m^2^.

The dried cast films were tested for tack and peel strength using an Instron 3000 Universal Tester, together with Bluehill 2 Materials Testing Software. Strips of 1′′×6′′ were cut for tack, and a drop loop was formed by taping 1′′ of each end of the strip, which was secured in the upper grip of the Instron. The upper grip was moved downward at a rate of 2 mm/s until an area of 1′′×1′′ of a stainless‐steel substrate mounted on the lower grip was covered. The upper grip was then pulled up at a 5 mm/s rate, and the maximum force (N/m) required for removal was recorded as tack.

Peel strength was measured by separating an adhesive from a substrate at a 180° angle with respect to the substrate. Strips of 1′′×5′′ were cut and adhered to a stainless‐steel substrate via application of a 2040 g roll coater (twice front‐to‐back movement along the length of the strip) at a rate of 10 mm/s. Subsequently, the substrate and the PSA strip were fixed in the lower and upper grips, respectively. The upper grip was then pulled upward at a rate of 5 mm/s, and the average force required for peeling (N/m) was reported as peel strength.

An in‐house built shear tester was used to record the time a PSA strip could hold against a constant vertical force. Strips of 1′′×5′′ were cut, and one end of the strip was laminated on a stainless‐steel substrate to cover a 1′′×0.5′′ area using the roll coater (2040 g) in a similar way to specimen preparation for the peel strength (four passes at a rate of 10 mm/s). A C‐clamp was used to hang a 500 g weight to the other end of the strip. The time (h) that passed until the covered area was removed and the hung weight dropped was reported as shear adhesion.

Each PSA test was repeated six times for each latex cast to film (three samples per film for each test), and the average results and standard deviations were recorded.

## Results and Discussions

**Figure 1:**
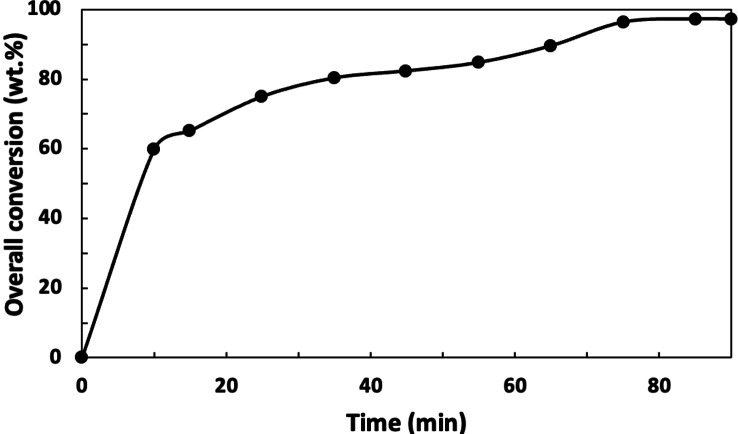
Typical overall monomer conversion versus time (BMV20 is shown as an example).

Using the formulation in Table 1, five polymerizations were conducted at BA/VAc ratios of 9 : 1 (w:w) with MDO concentrations varying from 0–20 wt.%. Overall monomer conversion versus time exhibited similar trends for all runs, culminating in final conversions exceeding 97 wt.% (Figure 1 and Table 2). The solids content ranged from 37–39 wt.%, with a target of 38 wt.% based on literature recommendations,[^20^, ^21^] which suggested reducing water content to minimize MDO hydrolysis. TGA analysis of the dried polymer samples was conducted to evaluate the presence of any residual small molecules, particularly MDO hydrolysis products. The thermogram showed no weight loss up to 350 °C (see Supporting Information, Figure S1), indicating no residual small molecules or volatile compounds in the dried polymer. Attempts to find direct evidence of MDO hydrolysis products, i. e., 4‐hydroxybutyl acetate (4‐HBA), were also made via water‐suppression ^1^H‐NMR spectroscopy on the final latex for each run. Unfortunately, the carbonyl groups for BA polymer and MDO polymer appear in the same range (3.5–4 ppm) as expected for 4‐HBA.^[21]^ Thus, the NMR results were inconclusive. However, due to the high monomer conversion levels, the TGA results, and the high MDO content in the polymer (see Table 2), we can conclude that hydrolysis was minimal if it did occur (see Supporting Information Figure S2 for additional details).

**Table 2:** Summary of final latex properties.

	Formulation		Final latex			
Latex ID^[a]^	BA/MDO/VAc monomer content (wt.%)	MDO monomer content (mol %)	Final conversion (wt.%)	MDO content^[b]^ (mol %)	Z‐average particle diameter (nm)	*T_g_* (°C)
**BV**	90/0/10	–	98.0±1	–	170.0±1.2	−50.24
**BMV5**	85.5/5.0/9.5	5.3	97.5±1	4.85	147.0±0.9	−49.79
**BMV10**	81.0/10.0/9.0	10.6	97.0±0.9	9.98	152.0±1.0	−47.25
**BMV15**	76.5/15.0/8.5	15.9	97.0±0.5	14.10	151.0±0.8	−37.45
**BMV20**	72.0/20.0/8.0	21.1	97.0±0.5	20.36	159.0±1.2	−34.49

^[a]^ Latex ID indicates monomer included in formulation (B=BA, M=MDO, V=VAc) and number refers to wt.% MDO. ^[b]^ MDO content represents the combined contribution of both ring‐opened and ring‐retained MDO.

The average particle size of the final latexes decreased after adding MDO but did not vary much with MDO content (Table 2). The final particle size distributions were narrow for all runs, exhibiting a low polydispersity index (PDI) of approximately 0.02. Data on the z‐average particle size versus time are shown in the Supporting Information. The polymer gel content also did not vary significantly, with an average value of 55 wt.%. As noted, it was impossible to filter the dissolved polymer (in THF) for molecular weight analysis. This was not surprising due to the high levels of BA in the formulation.^[25]^

**Figure 2:**
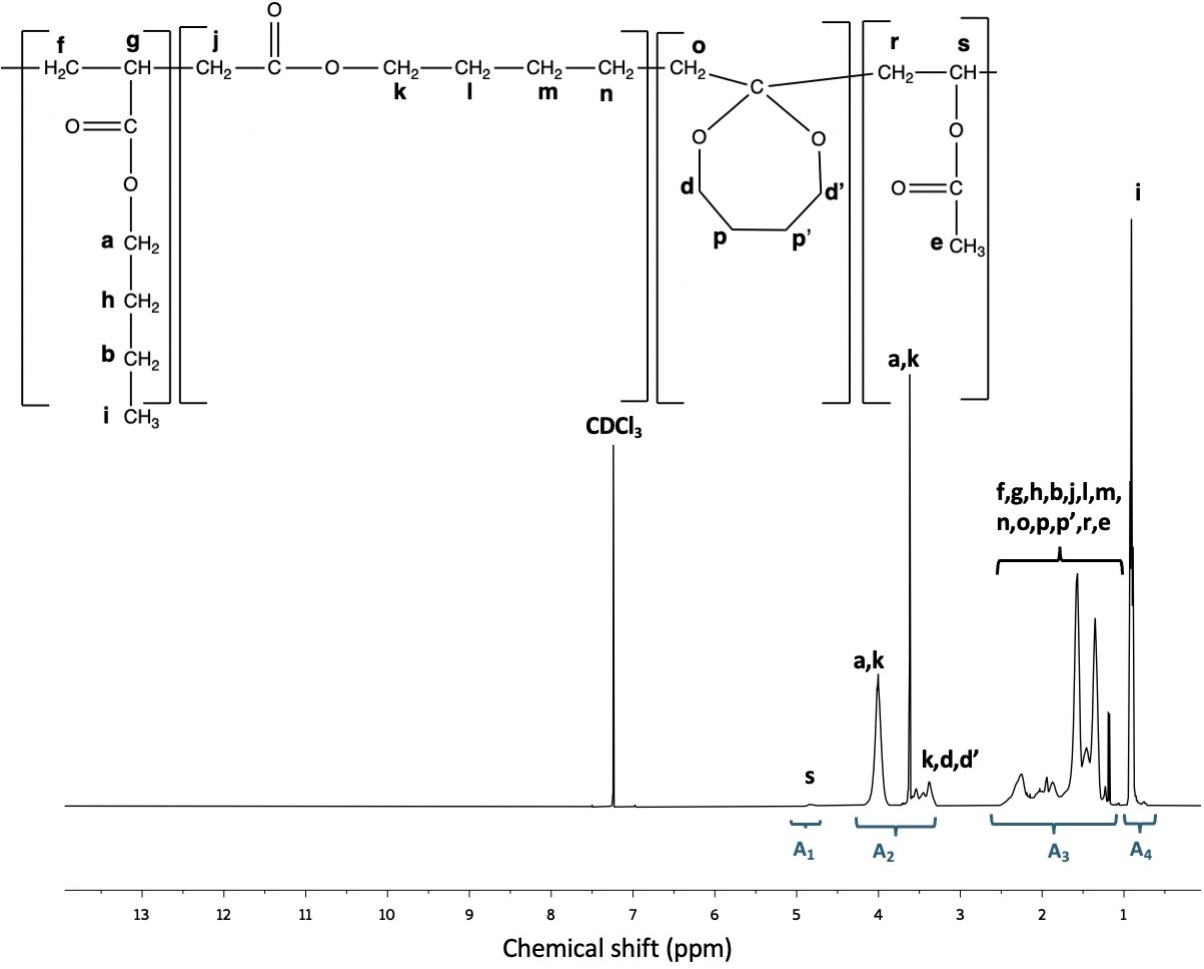
^1^H‐NMR spectrum for BMV20 in CDCl_3_. Detailed peak assignments are shown in the Supporting Information (Figure S5).

The MDO content in the final latex was calculated from ^1^H‐NMR spectroscopy (Figure 2) and ranged from 4.45–20.36 mol % (Table 2). The analysis was like that used previously.^[14]^ The composition was calculated from four regions, A_1_, A_2_, A_3_ and A_4_, shown in Figure 2. Area A_1_ represents the −CH protons of VAc (δ=4.7–4.9 ppm), area A_2_ represents the −CH_2_O protons of BA and MDO (δ=3.3–4.2 ppm), area A_4_ represents the −CH_3_ of BA (δ=0.7–1 ppm) and area A_3_ encompasses the remaining protons for BA, MDO, and VAc (δ=1–2.5 ppm). It should be noted that there were some overlapping peaks for peak assignments “a, k” and “k, d’, d”. In those runs conducted at 40 °C, the percentage of MDO retained in its ring structure was approximately 27 mol % for BMV5, 26 mol % for BMV10, 30 mol % for BMV15, and 30 mol % for BMV20. The calculation method, along with detailed peak assignments and the ^1^H‐NMR spectra for the other runs, are provided in the Supporting Information (Figures S4–S8).

**Figure 3:**
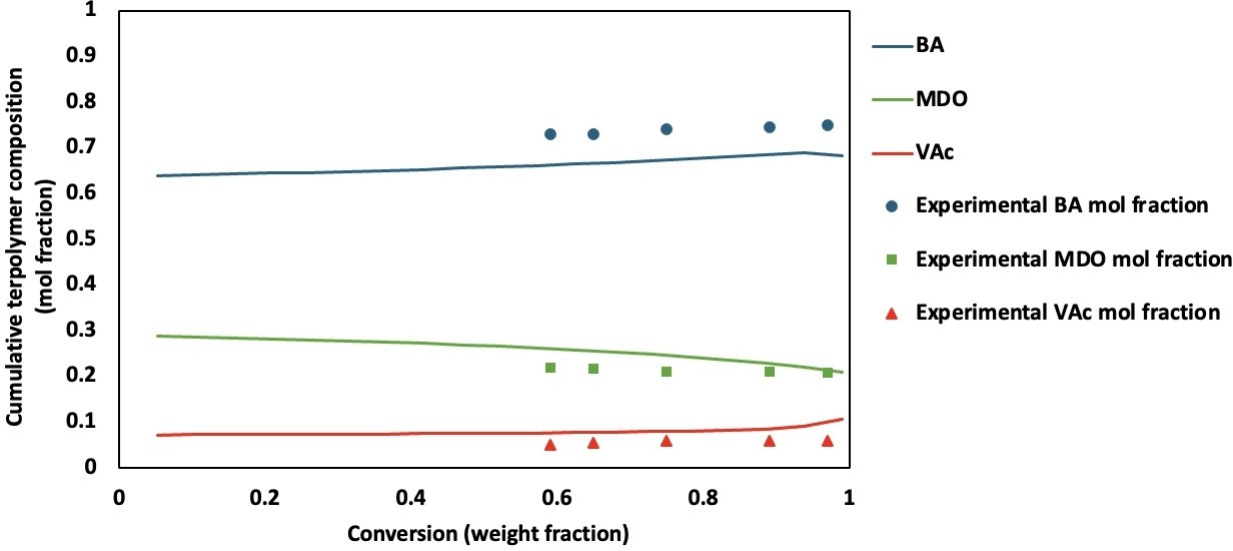
Model predictions of cumulative terpolymer composition using ternary reactivity ratios (solid lines) for BMV20. Experimental data are shown as symbols for BA (blue circles), MDO (green squares) and VAc (red triangles). Additional figures for other feed compositions are shown in the Supporting Information.

**Figure 4:**
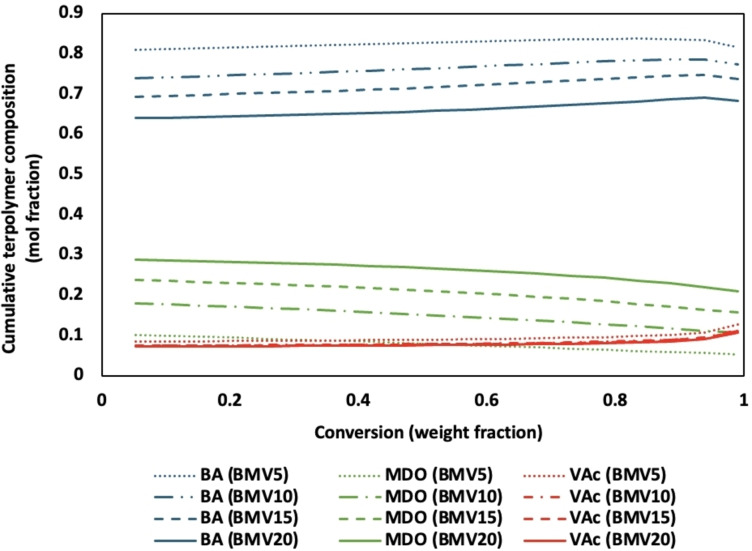
Model predictions of cumulative terpolymer composition vs conversion using ternary reactivity ratios for all terpolymers (BMV5, BMV10, BMV15, and BMV20).

Analysis of the terpolymer composition versus conversion revealed a homogeneous distribution of MDO throughout the polymer backbone, which is crucial for enhancing the final product′s degradation properties (Figure 3 and Supporting Information).^[11]^ The nearly constant amount of MDO throughout the polymerization indicates a uniform distribution of MDO in each composition. The ternary reactivity ratios for the system (BA/MDO/VAc, 1/2/3) were previously determined using an integrated error‐in‐variables method to be r_12_=0.417, r_21_=0.071, r_13_=4.459, r_31_=0.198, r_23_=0.260, and r_32_=55.339,^[14]^ and predict a uniform distribution of MDO for all feed compositions studied (Figure 4). It should be noted that the model predictions are for bulk polymerization, and no correction for partitioning of monomer in the water phase was accounted for. Nevertheless, the model predictions of the terpolymer composition are excellent (Figure 3 and Supporting Information). The reactivity ratios suggest a preferential reaction of MDO with BA and VAc, supporting the observed compositional homogeneity. Furthermore, the DSC results of all compositions (see Supporting Information) showed a single inflection point, which points to the formation of random terpolymers with a uniform distribution of MDO units. This random distribution of MDO within the terpolymer matrix has significant implications for the properties of the dried latex, particularly its degradability and compostability. A uniform distribution of MDO ensures that the degradable linkages are evenly dispersed throughout the polymer, promoting consistent and efficient degradation when exposed to environmental conditions conducive to composting. However, due to the presence of ring‐retained MDO in the terpolymers, these four runs (Table 2) would not necessarily have uniformly distributed MDO ester groups in the terpolymer backbone.

**Scheme 1:**
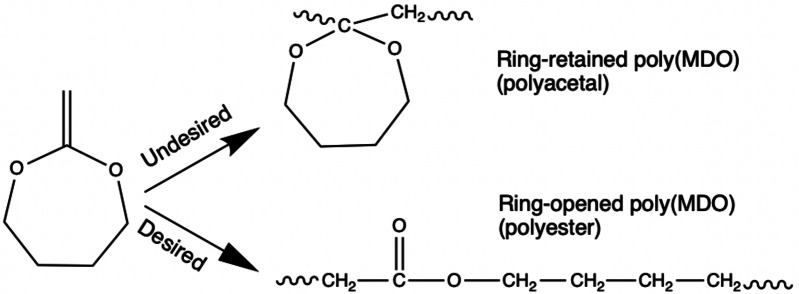
Free radical polymerization of MDO leads to ring‐retained MDO (polyacetal) or ring‐opened MDO (polyester).

**Figure 5:**
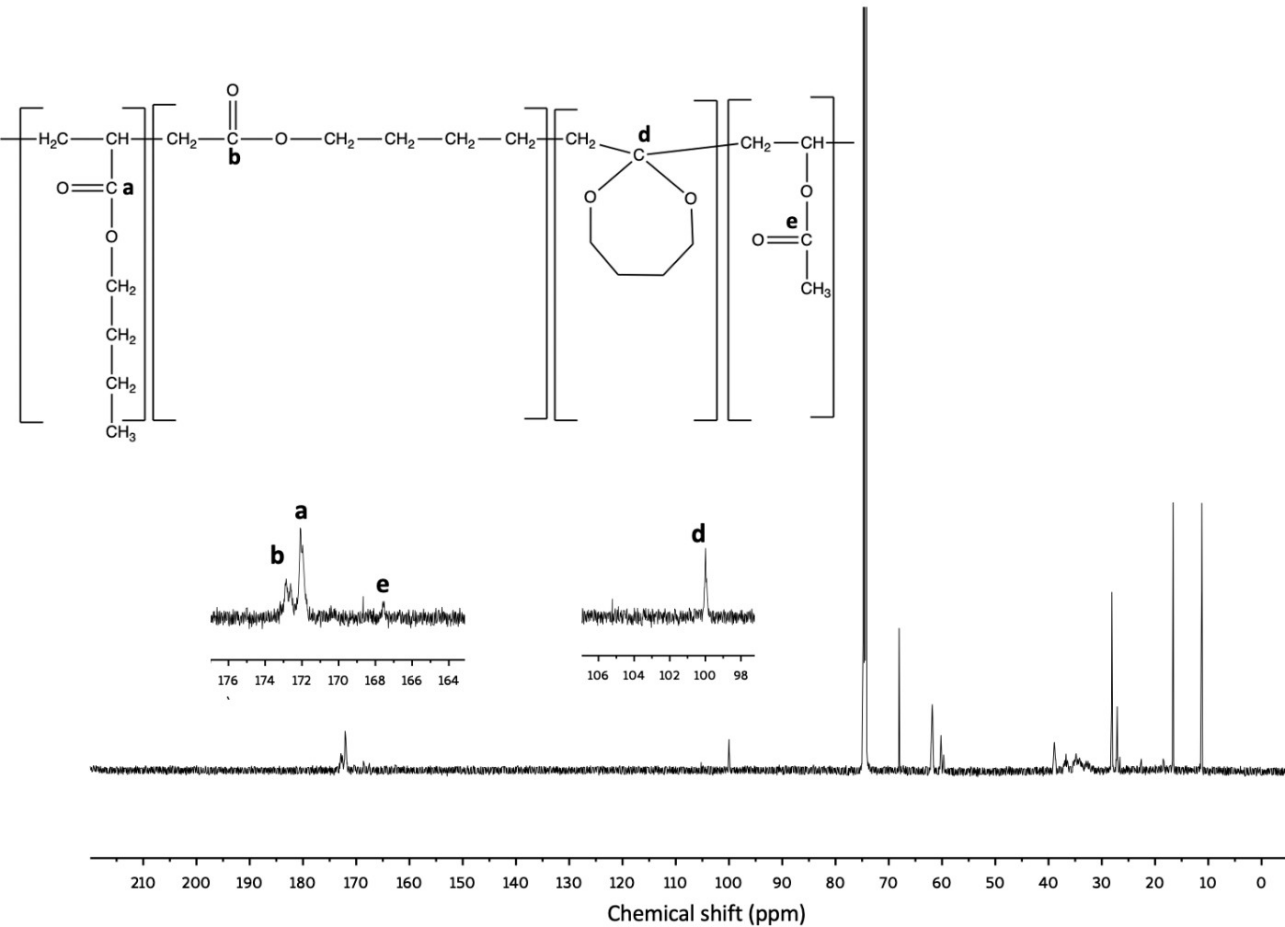
^13^C‐NMR spectrum for BMV20 in CDCl_3_. The peak at 105 ppm is a narrow artifact observed in our ^13^C‐NMR results using the AVIII600 NMR instrument.

It is well known that the free‐radical polymerization of MDO can result in various products.^[42]^ These possibilities include complete ring‐opening leading to polyester formation, a mix of ring‐opening and polyacetal formation (i. e., due to ring retaining polymerization), or complete polyacetal formation (Scheme 1). The specific outcome depends on factors such as the comonomer, initiator type, and polymerization temperature.[^42^, ^43^] As noted earlier, results from ^1^H‐NMR spectra raised doubts in determining the precise amount of ring‐retained MDO due to peak overlap. Quantitative ^13^C‐NMR spectra were then used to determine the proportion of ring‐retained MDO units in the terpolymer. In Figure 2, the ^13^C‐NMR results for BMV20 show peaks at approximately 167.5, 172, and 173 ppm, related to the −COO groups of VAc (“e”), BA (“a”), and MDO ring‐opened polymerization (“b”), respectively. The peak at ~100 ppm, indicates the cyclic structure of ring‐retained MDO polymerization (shown as “d”). The analysis suggested that the ring‐retained MDO content in the terpolymer ranged from 15–50 mol % of the MDO polymerized; however, due to the low signal‐to‐noise ratio typical of ^13^C‐NMR spectra, the reliability of the quantities is in question compared to the values calculated via ^1^H‐NMR shown earlier. Nevertheless, a visible peak at 100 ppm (peak “d”, Figure 5) suggests significant ring‐retained MDO in the terpolymers. Spectra and calculations for the other three runs are shown in the Supporting Information (Figures S11–S13).

Attempts to measure the proportion of ring‐retained MDO via DSC gave results ranging from 3–15 wt.%. Literature suggests that ring‐opened MDO homopolymer typically exhibits a *T_g_* of approximately −59 °C, lower than BA homopolymer at −54 °C. Consequently, it was anticipated that incorporating ring‐opened MDO would reduce the terpolymer′s *T_g_*. However, our experimental data showed a contrasting trend (Table 2). Analysis suggested that the formation of polyacetal structures (i. e., ring‐retained MDO) rather than polyester plays a critical role in the observed *T_g_* behavior. Polyacetal chains are known for their rigidity due to their cyclic nature, leading to reduced segmental mobility and higher *T_g_*. To further investigate the *T_g_* behavior, the Fox equation was employed to calculate the theoretical weight fraction of ring‐retained MDO in the terpolymer:

$$\frac{1}{T_{g_{total}}}=\frac{w_{BA}}{T_{g_{BA}}}+\;\frac{w_{VAc}}{T_{g_{VAc}}}+\;\frac{w_{MDO(ring-opened)}}{T_{g_{MDO\;(ring-opened)}}}+\;\frac{w_{MDO(ring-retained)}}{T_{g_{MDO\;(ring-retained)}}}$$

where *w_BA_*, *w_VAc_*, *w*_*MDO(ring‐opened)*_, *w*_*MDO(ring‐retained)*_ are the weight fractions of BA, VAc, ring‐opened MDO, and ring‐retained MDO, respectively. The *T_g_* values used in the equation were $T_{g_{BA}}$ =−54 °C, $T_{g_{VAc}}$ =30 °C, $T_{g_{MDO\;(ring-opened)}}$ =−59 °C, and $T_{g_{MDO\;(ring-retained)}}$ ≈183 °C. The weight fractions of BA, VAc, and total MDO (including both ring‐opened and ring‐retained forms) were employed from the ^1^H‐NMR peak assignments, while the overall *T_g_s* were obtained from DSC results. Although widely used, the Fox equation is not entirely reliable for copolymer or terpolymer *T_g_* estimation.^[44]^ The calculated ring‐retained MDO content using the Fox equation ranged from 3–15 wt.%. However, these values were significantly lower than the ~40 wt.% ring‐retained MDO calculated from the quantitative ^13^C‐NMR.

Given the uncertainty in the *T_g_* for ring‐retained MDO, the unreliability of the Fox equation, and the low signal‐to‐noise ratio of the ^13^C‐NMR spectra, providing a reliable measure of the amount of ring‐retained MDO is not possible. However, as noted earlier, there is no doubt from both the DSC and ^13^C‐NMR results that significant ring‐retained MDO is present in the terpolymers.

Table 3 presents the comparative results of PSAs with varying MDO content. Based on commercial PSAs[^38^, ^45^] and our previous lab‐made adhesives[^38^, ^46^] the tack and shear adhesion values were within an acceptable range; however, the peel strength was low. The initial castability of our PSA was low due to the low solids content in the latexes, necessitating the addition of a wetting agent to improve film formation (see Supporting Information). Without the wetting agent, it was challenging to form a uniform film. Once the wetting agent was added, uniform films were produced. The addition of a wetting agent, however, can lead to poorer PSA performance.^[25]^ The inclusion of 5 and 10 wt.% MDO did not significantly alter the tack and peel strength, indicating a threshold effect on the influence of MDO. However, when the concentration was increased to 15 and 20 wt.%, there was a decrease in tack and peel strength. Shear adhesion was found to decrease with the addition of MDO at 5 wt.% initially but increased with further increases in MDO concentration. The decline in tack and peel strength with higher MDO content can be attributed to the increased stiffness of the adhesive film, which diminished its ability to effectively wet and bond to surfaces.^[25]^ Thus, the effect was due to the increase in *T_g_* (Table 2) at higher MDO levels, reflecting the augmented rigidity of the polymer matrix. The increased *T_g_* also contributed to the increased shear adhesion.

**Table 3:** PSA properties of dried cast films.

Latex ID	Tack (N/m)	Peel strength (N/m)	Shear adhesion (h)
**BV**	210±15	49±7	3±1
**BMV5**	208±11	49±6	2.2±0.5
**BMV10**	210±10	47±7	2.8±1
**BMV15**	190±10	25±5	3.2±1
**BMV20**	175±9	25±5	4±1.2

**Figure 6:**
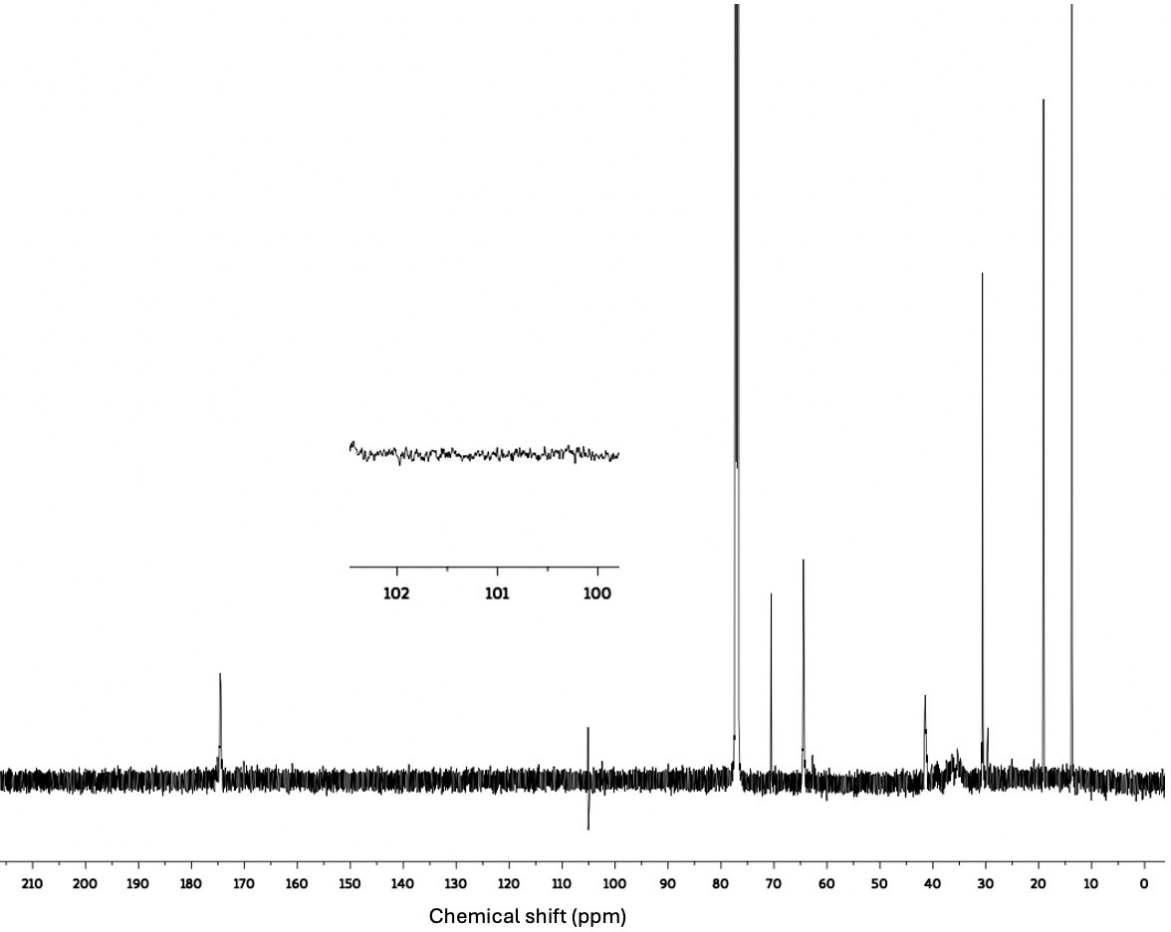
^13^C‐NMR spectrum for BMV10‐NEW in CDCl_3_. This result is without the addition of cCNCs. The peak at 105 ppm is a narrow artifact observed in our ^13^C‐NMR results using the AVIII600 NMR instrument.

To mitigate MDO ring retention and enhance PSA properties without causing MDO hydrolysis, an additional run was conducted by increasing the polymerization temperature to 50 °C. The solids content was increased to 45 wt.% to decrease the possibility of MDO hydrolysis.^[21]^ Finally, a cCNC suspension was added to the final latex via blending to improve the PSA properties.[^38^, ^45^, ^46^] For the additional run, the polymer composition of terpolymer BMV10 was used. The research literature suggests that adding 5–10 mol % MDO to the polymer backbone is a practical starting point for achieving biodegradation.^[14]^ The new BMV10 terpolymer latex (i. e., BMV10‐NEW) had a solids content of ~45 wt.% before adding the cCNC suspension, while it decreased to ~38 wt.% after the cCNC suspension was added. Monomer conversion was almost 97 wt.%. However, due to the increased solids content, the polymer particle size increased by ~130 nm–280 nm (see Supporting Information for more details). Based on the ^1^H‐NMR results (see Supporting Information), the MDO content was 8.5 mol %, and our ^1^H‐NMR calculations indicated approximately 9 mol % of the MDO was retained in its ring structure. However, the ^13^C‐NMR results did not show any peak at 100 ppm. As shown in the ^13^C‐NMR spectrum in Figure 6, there was no peak at 100 ppm, indicating that no MDO ring retention was detectable. Additionally, DSC results showed a decreased terpolymer *T_g_* from −47.25 °C (for the original BMV10) to −52.6 °C, which aligns with the MDO predominantly undergoing ring‐opening polymerization.

As noted above, 2.5 wt.% of a never‐dried cCNC suspension in water was added to the latex at the end of the polymerization via blending to further enhance the PSA properties. The never‐dried cCNC suspension (6.6 wt.%) was diluted in DDW to 2.5 wt.%, mixed for about 5 minutes with a magnetic stir bar, and then ultrasonicated before being added to the BMV10‐NEW latex. Probe ultrasonication of the cCNC suspension was performed in an ice bath using a Fisher Scientific 550 sonic dismembrator at 75 % amplitude for three intervals of 5 minutes, with 5 minute rest periods in between. The cCNC suspension was blended into the latex in the reactor at the polymerization temperature (50 °C) at a reactor mixing speed of 300 rpm. According to our previous work, these blending conditions are optimal for simultaneously enhancing all PSA properties.^[45]^ Because of the addition of the water from the cCNC suspension, the final solids content prior to film casting was comparable to the previous runs; the dried film thicknesses were measured and shown to be the same. The BMV10‐NEW‐cCNC dried latex films showed no deficiencies, were smooth and free of holes, and no wetting agent was needed (see Supporting Information). Table 4 presents the PSA properties after adding the cCNCs. All PSA properties increased simultaneously after polymerizing at 50 °C and adding the cCNCs, with peel strength and shear adhesion increasing by a factor of almost 2 compared to the original BMV10 latex films.

**Table 4:** PSA properties of the cast films.

Latex ID	Tack (N/m)	Peel strength (N/m)	Shear adhesion (h)
**BMV10**	210±10	47±7	2.8±1
**BMV10‐NEW‐cCNC** ^[a]^	280±15	120±10	6.2±1.5

^[a]^ BMV10‐NEW‐cCNC represents the new BMV10 run incorporating cCNC.

## Conclusions

The synthesis and characterization of BA/MDO/VAc emulsion terpolymers, focusing on the innovative use of MDO in emulsion polymerization for PSA applications, has been demonstrated. By increasing the polymerization temperature to 50 °C and optimizing the solids content, we effectively promoted MDO ring‐opening while controlling hydrolysis. Water suppression, ^1^H‐NMR spectroscopy, high monomer conversion levels, and the polymer composition led us to conclude that MDO hydrolysis was minimized if it was present. In addition, the DSC and ^13^C‐NMR spectroscopy confirmed the successful incorporation of MDO with no ring retention. The ^1^H‐NMR spectroscopy of samples throughout each run showed that a batch polymerization approach was sufficient to enable the even distribution of MDO in the polymer chains. This was not surprising, given the reactivity ratios for this system. The key to controlling MDO hydrolysis was maintaining a pH above neutral in the reaction mixture, minimizing the exposure to water, and using a relatively low reaction temperature. On the other hand, hydrolysis of VAc was a concern (at higher pH), and a sufficiently elevated reaction temperature was necessary to minimize MDO ring retention. The addition of cCNCs enabled the production of PSA films with significantly improved properties, particularly peel strength and shear adhesion. This study underscores the potential of using bio‐based monomers and green chemistry principles to create degradable, high‐performance adhesive materials for PSA applications.

## Conflict of Interests

The authors declare no conflict of interest.

1

## Supporting information

As a service to our authors and readers, this journal provides supporting information supplied by the authors. Such materials are peer reviewed and may be re‐organized for online delivery, but are not copy‐edited or typeset. Technical support issues arising from supporting information (other than missing files) should be addressed to the authors.

Supporting Information

## Data Availability

The data that support the findings of this study are available from the corresponding author upon reasonable request.
