# Supplementary material for: Butyl Acrylate/2‐Methylene‐1,3‐Dioxepane/Vinyl Acetate Emulsion Terpolymerization: Incorporating Backbone Degradable Linkages into Adhesive Applications
Source: ChemSusChem. 2025 Jan 8;18(9):e202402478. doi: 10.1002/cssc.202402478 (PMC12051237; doi:10.1002/cssc.202402478)
Supplement: Supplementary file 1 — Supporting Information [file CSSC-18-e202402478-s001.pdf]

ChemSusChem

Supporting Information

# Butyl Acrylate/2-Methylene-1,3-Dioxepane/Vinyl Acetate Emulsion Terpolymerization: Incorporating Backbone Degradable Linkages into Adhesive Applications

Maryam Movafagh, Kelly M. Meek,* and Marc A. Dubé

Supporting information for

# Butyl Acrylate/2-Methylene-1,3-Dioxepane/Vinyl Acetate Emulsion Terpolymerization: Incorporating Backbone Degradable Linkages into Adhesive Applications

Authors

Maryam Movafagh[1], Kelly M. Meek[1,2*], Marc A. Dubé[1]

[1]Department of Chemical and Biological Engineering, University of Ottawa, Ottawa, ON

[2]Department of Chemical and Materials Engineering, Concordia University, Montreal, QC

[*] to whom correspondence should be addressed: kelly.meek@concordia.ca

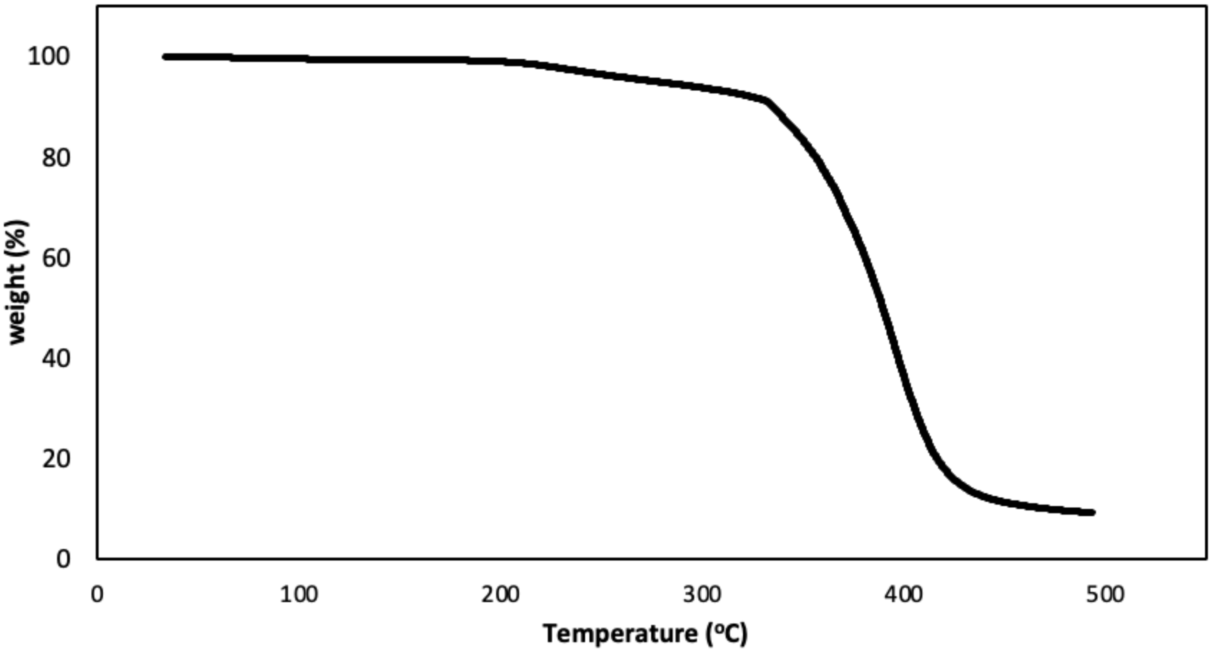


Figure S1. TGA results of BMV20 after drying for solids content measurement

Scheme S1. General scheme of MDO hydrolysis to 4-hydroxybutyl acetate (4-HBA)

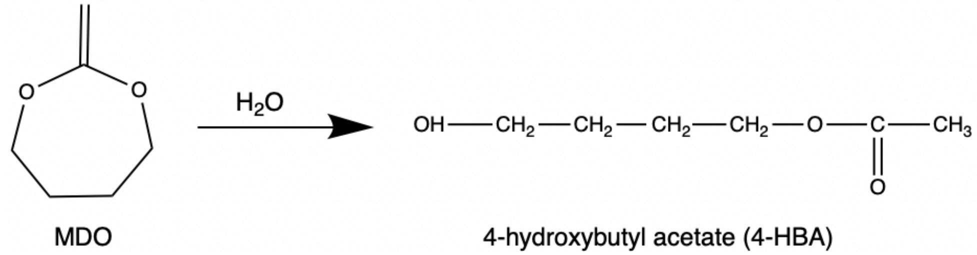

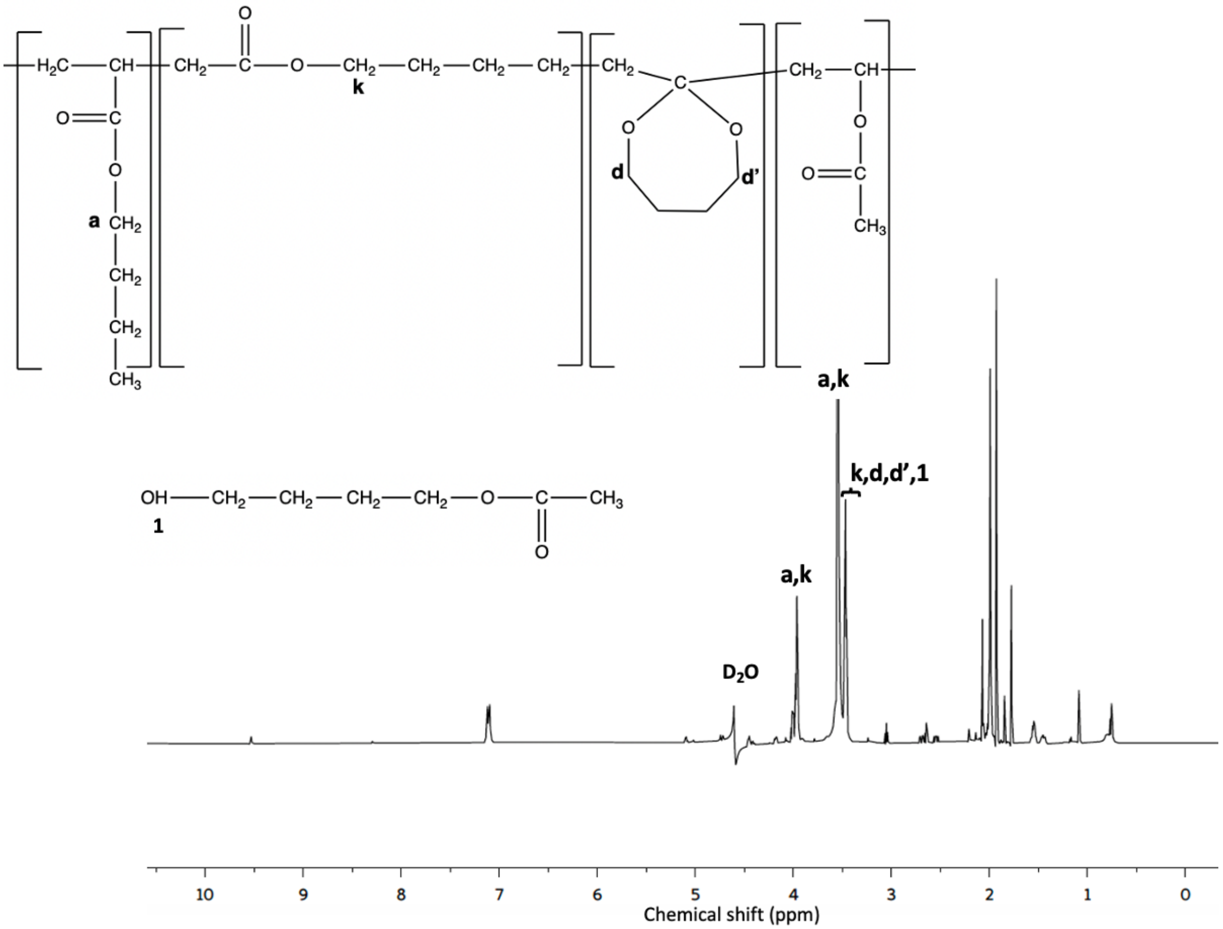


Figure S2. $^1$H-NMR Watergate spectrum of BMV20 latex in $D_2O$.

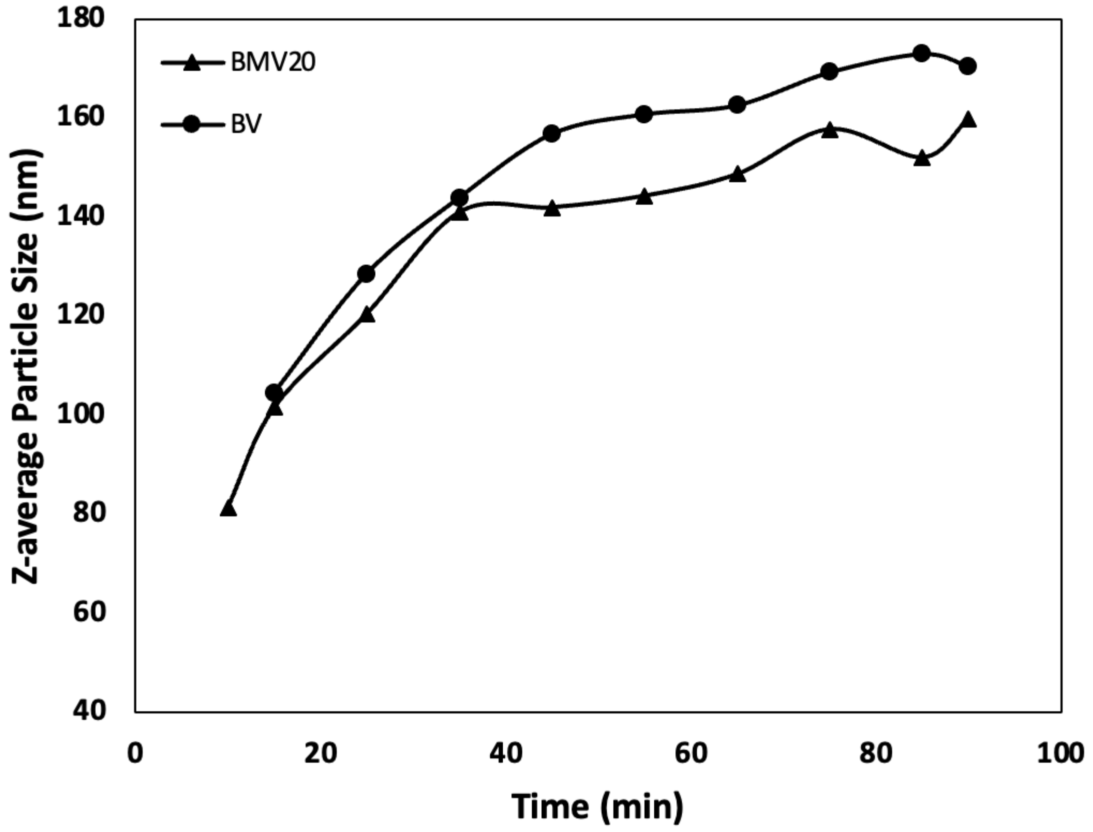


Figure S3. Z-average particle size versus time for BV and BMV20. All terpolymers—BMV5, BMV10, BMV15, and BMV20—exhibit similar particle sizes and follow the same trend. BMV20 is used here as a representative example for comparison with BV.

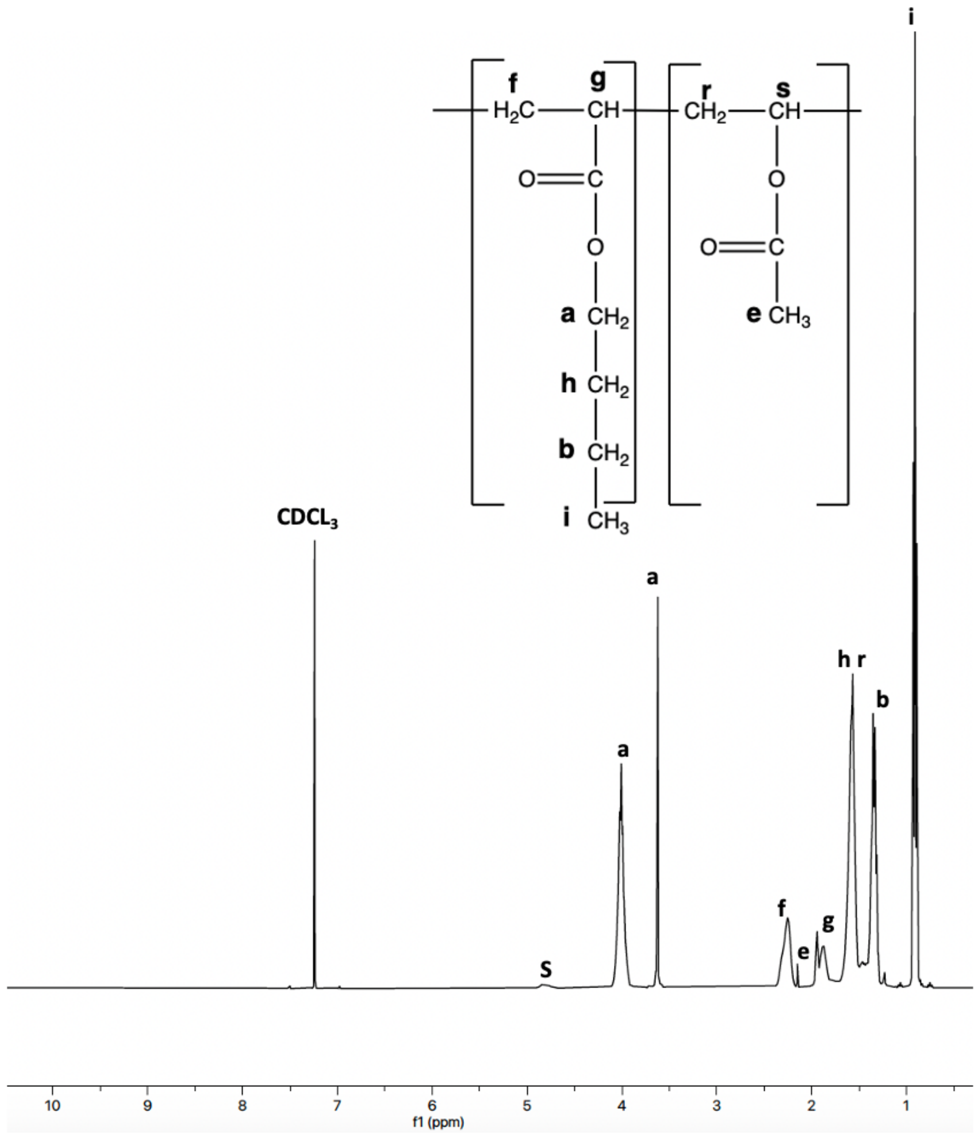


Figure S4. $^{1}$H-NMR spectrum for BV in $CDCl_3$.

The BA/MDO/VAc terpolymer compositions were calculated using the following equations:

$$A_1 = H_{VAc} \quad (1)$$

$$A_2 = 2H_{BA} + 2H_{MDO\text{-}ro} + 4H_{MDO\text{-}rr} \quad (2)$$

$$A_3 = 3H_{BA} \quad (3)$$

$$A_4 = 7H_{BA} + 8H_{MDO\text{-}ro} + 6H_{MDO\text{-}rr} + 5H_{VAc} \quad (4)$$

$$F_{\mathrm{BA}} = H_{BA} / (H_{BA} + H_{MDO\text{-}ro} + H_{MDO\text{-}rr} + H_{VAc}) \quad (5)$$

$$F_{\mathrm{MDO\text{-}ro}} = H_{MDO\text{-}ro} / (H_{BA} + H_{MDO\text{-}ro} + H_{MDO\text{-}rr} + H_{VAc}) \quad (6)$$

$$F_{\mathrm{MDO\text{-}rr}} = H_{MDO\text{-}rr} / (H_{BA} + H_{MDO\text{-}ro} + H_{MDO\text{-}rr} + H_{VAc}) \quad (7)$$

$$F_{\mathrm{VAc}} = H_{VAc} / (H_{BA} + H_{MDO\text{-}ro} + H_{MDO\text{-}rr} + H_{VAc}) \quad (8)$$

where $H_{BA}$, $H_{MDO\text{-}ro}$, $H_{MDO\text{-}rr}$, $H_{VAc}$ represent the BA, ring-opened MDO, ring-retained MDO and VAc protons, respectively, found in areas $A_1$, $A_2$, $A_3$, and $A_4$. Area $A_1$ represents the −CH protons of VAc (δ = 4.7 – 4.9 ppm), $A_2$ represents the $-CH_2O$ protons of BA and MDO (δ = 3.3 – 4.2 ppm), area $A_4$ represents the $-CH_3$ protons of BA (δ = 0.7 – 1 ppm), and $A_3$ encompasses the remaining protons for BA, MDO, and VAc (δ = 1 – 2.5 ppm). After solving for $H_{BA}$, $H_{MDO\text{-}ro}$, $H_{MDO\text{-}rr}$, and $H_{VAc}$ (Equations 1, 2, 3, and 4), the terpolymer compositions were calculated using Equations 5, 6, 7, and 8. This composition analysis was conducted for all the BA/MDO/VAc terpolymers. For example, for the BMV20 terpolymer, the integration values were obtained as follows $A_1 = 1$, $A_2 = 42.46$, $A_3 = 115.16$, $A_4 = 47.11$; using these values in Equations 1–4, we solved for $H_{BA}$, $H_{MDO\text{-}ro}$ $H_{MDO\text{-}rr}$, and $H_{VAc}$. Substituting $H_{BA}$, $H_{MDO\text{-}ro}$ $H_{MDO\text{-}rr}$, and $H_{VAc}$ into Equations 5–8, the molar fractions for each component in BMV20 were determined as $F_{\mathrm{BA}} = 0.75$, $F_{\mathrm{MDO}} = 0.20$ ($F_{\mathrm{MDO\text{-}ro}} = 0.14$, $F_{\mathrm{MDO\text{-}rr}} = 0.06$), and $F_{\mathrm{VAc}} = 0.05$. This calculation process was applied consistently for all other runs, with each run's MDO contents reported in Table 2 of the main manuscript.

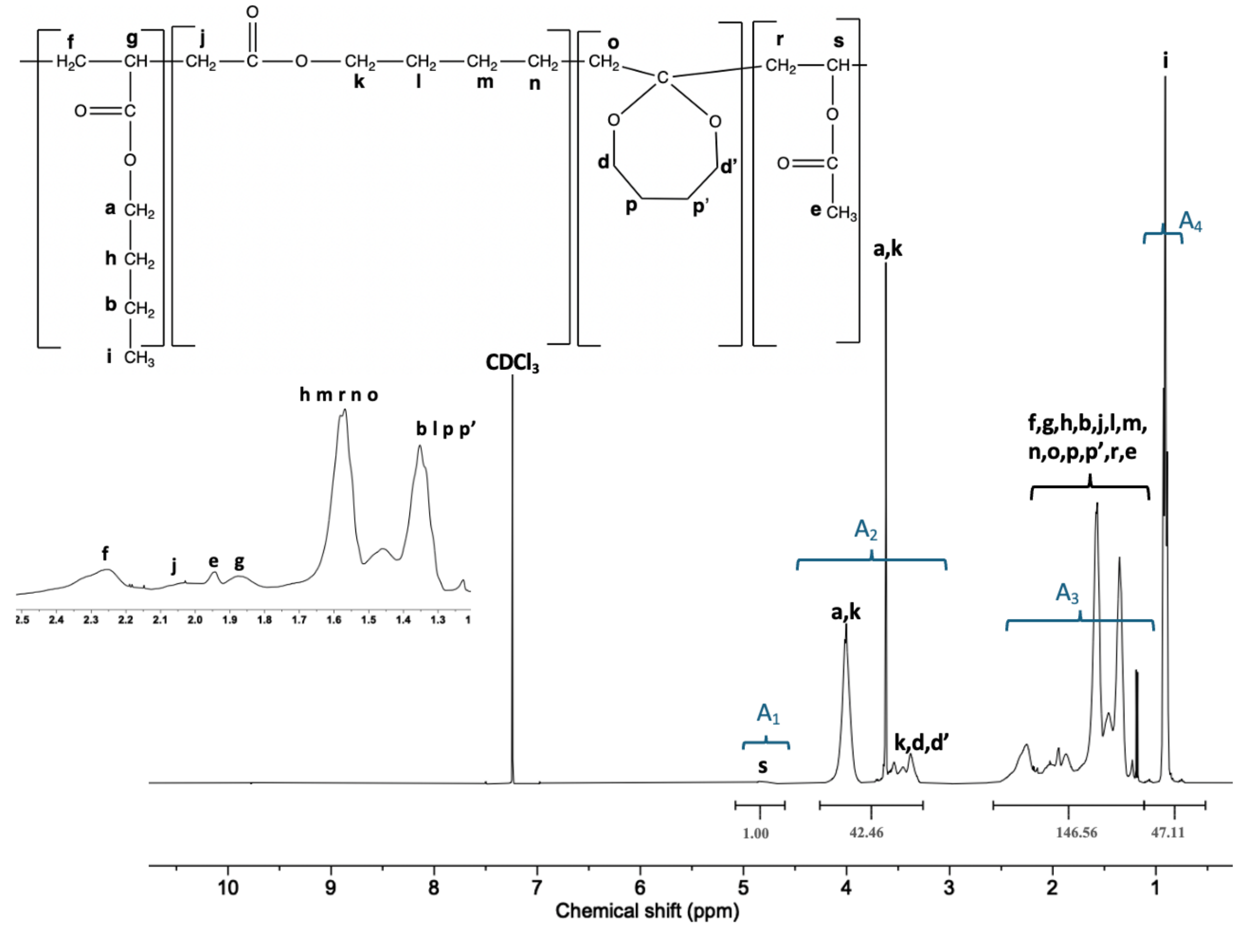


Figure S5. ¹H-NMR spectrum of BMV20 in $CDCl_3$, shows peak assignments and integration values for protons corresponding to BA, MDO (both ring-opened and ring-retained forms), and VAc.

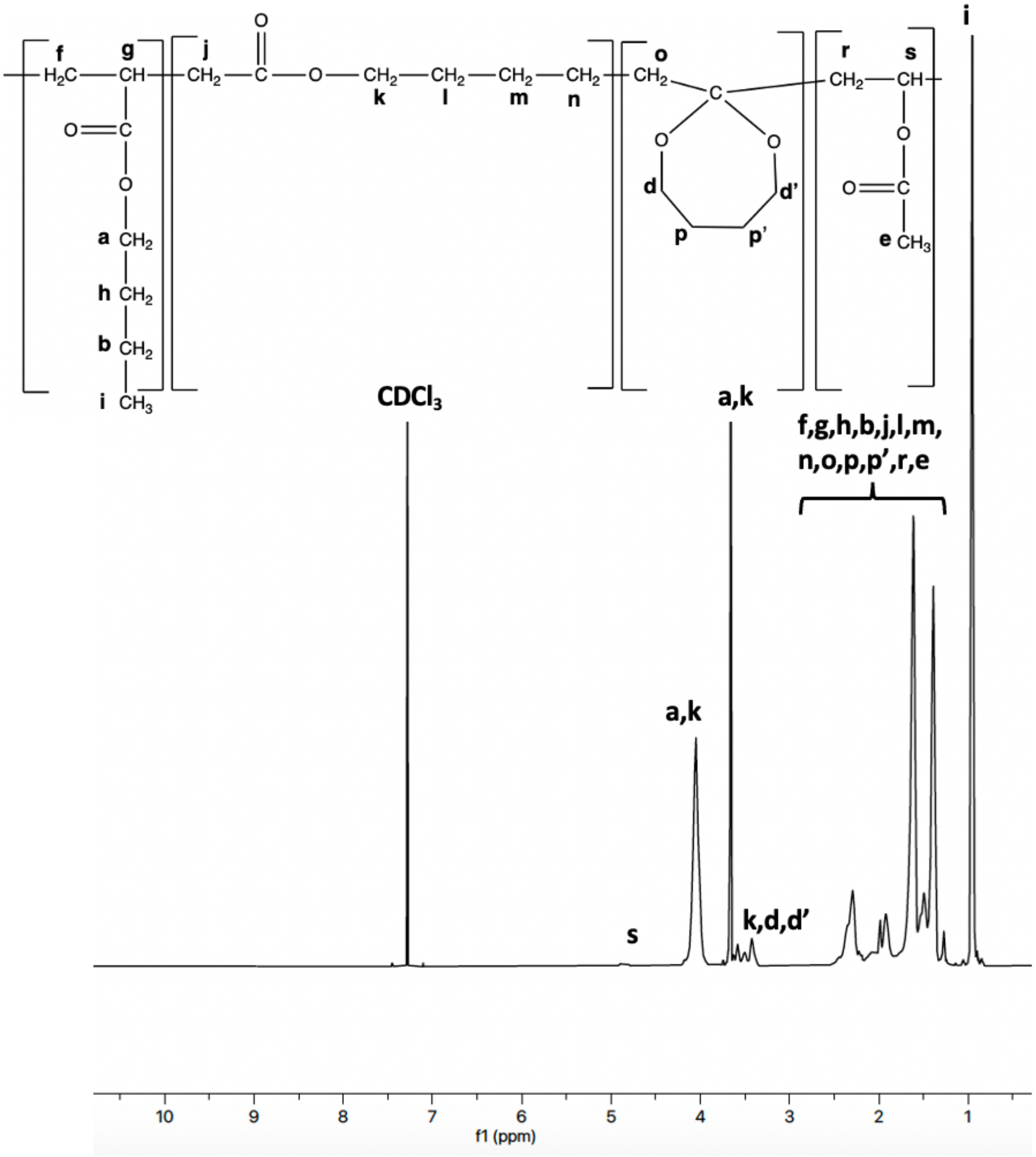


Figure S6. $^1$H-NMR spectrum for BMV5 in $CDCl_3$. See Figure S5 for detailed peak assignments.

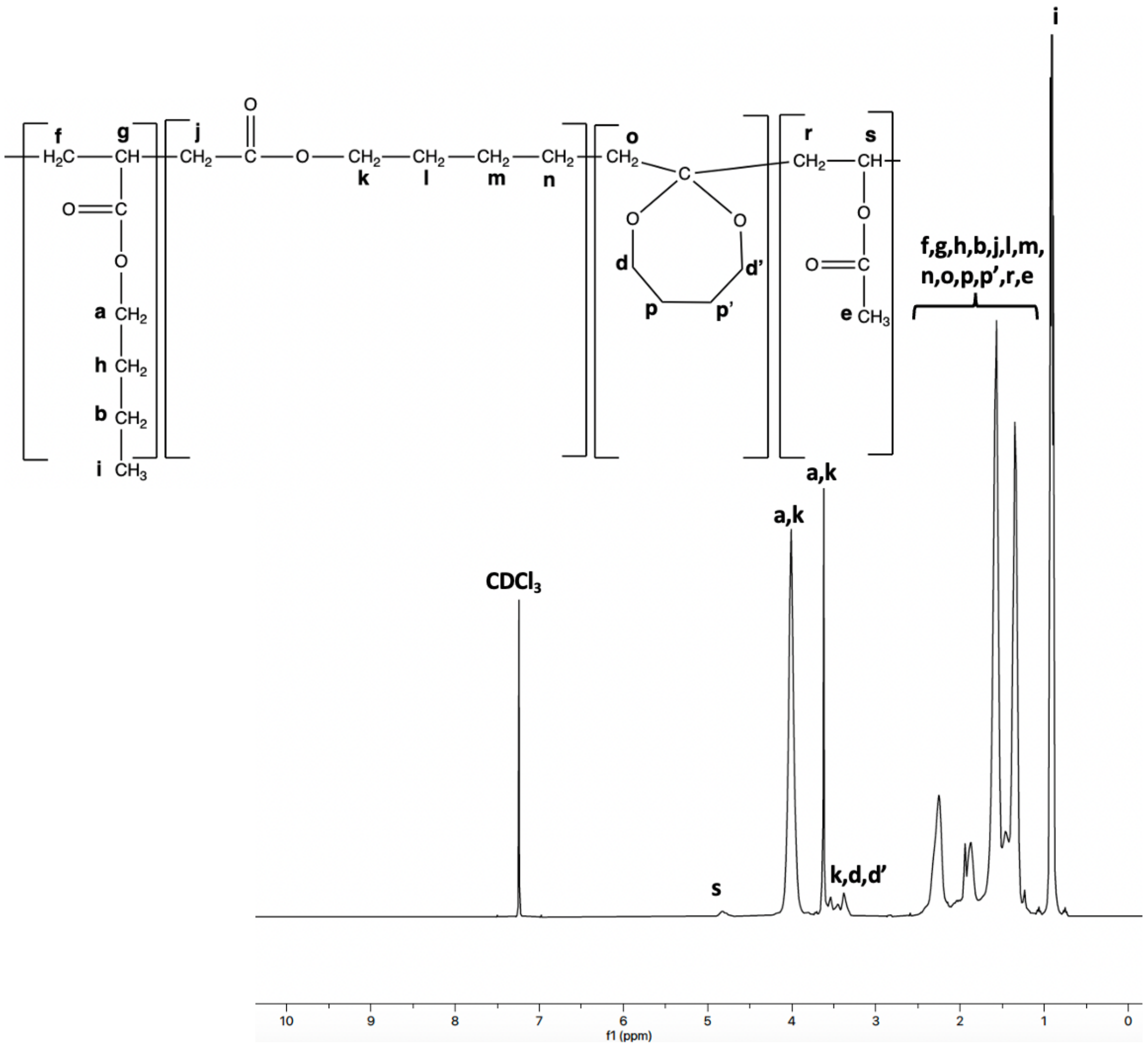


Figure S7. $^1$H-NMR spectrum for BMV10 in $CDCl_3$. See Figure S5 for detailed peak assignments.

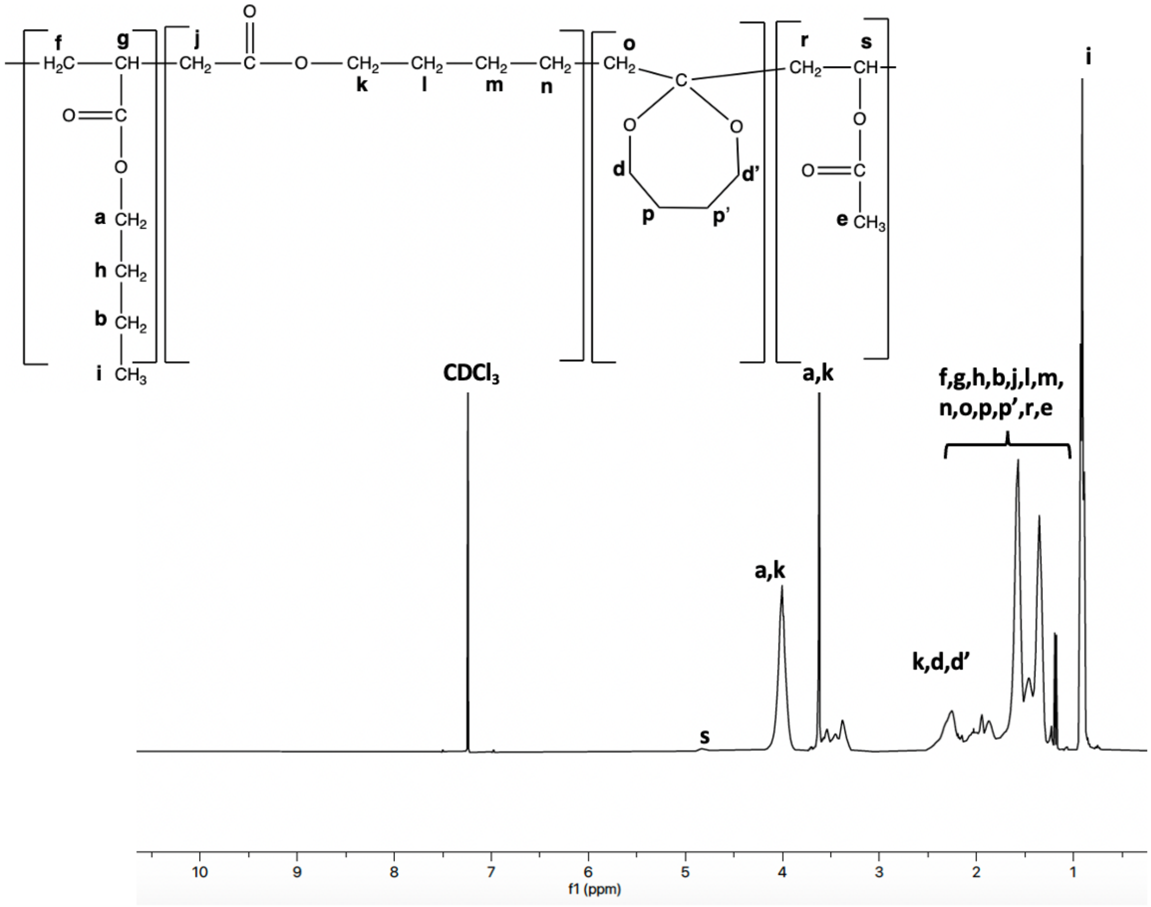


Figure S8. $^1$H-NMR spectrum for BMV15 in $CDCl_3$. See Figure S5 for detailed peak assignments.

Equations 9–11 (see below) predict the terpolymer compositions without the complexities of ratio-based responses. Here, $F_i$ represents the instantaneous mole fraction of monomer $i$ bound in the terpolymer, $f_i$ represents the mole fraction of unreacted monomer $i$ in the polymerizing mixture, and the reactivity ratios are represented as $r_{ij}$.

$$F_1 - \frac{f_1\left(\frac{f_1}{r_{21}r_{31}}+\frac{f_2}{r_{21}r_{32}}+\frac{f_3}{r_{31}r_{23}}\right)\left(f_1+\frac{f_2}{r_{12}}+\frac{f_3}{r_{13}}\right)}{\left(\begin{array}{c} f_1\left(\frac{f_1}{r_{21}r_{31}}+\frac{f_2}{r_{21}r_{32}}+\frac{f_3}{r_{31}r_{23}}\right)\left(f_1+\frac{f_2}{r_{12}}+\frac{f_3}{r_{13}}\right) \\ +f_2\left(\frac{f_1}{r_{12}r_{31}}+\frac{f_2}{r_{12}r_{32}}+\frac{f_3}{r_{13}r_{32}}\right)\left(f_2+\frac{f_1}{r_{21}}+\frac{f_3}{r_{23}}\right) \\ +f_3\left(\frac{f_1}{r_{13}r_{21}}+\frac{f_2}{r_{23}r_{12}}+\frac{f_3}{r_{13}r_{23}}\right)\left(f_3+\frac{f_1}{r_{31}}+\frac{f_2}{r_{32}}\right) \end{array}\right)} = 0 \tag{9}$$

$$F_2 - \frac{f_2\left(\frac{f_1}{r_{12}r_{31}}+\frac{f_2}{r_{12}r_{32}}+\frac{f_3}{r_{13}r_{32}}\right)\left(f_2+\frac{f_1}{r_{21}}+\frac{f_3}{r_{23}}\right)}{\left(\begin{array}{c} f_1\left(\frac{f_1}{r_{21}r_{31}}+\frac{f_2}{r_{21}r_{32}}+\frac{f_3}{r_{31}r_{23}}\right)\left(f_1+\frac{f_2}{r_{12}}+\frac{f_3}{r_{13}}\right) \\ +f_2\left(\frac{f_1}{r_{12}r_{31}}+\frac{f_2}{r_{12}r_{32}}+\frac{f_3}{r_{13}r_{32}}\right)\left(f_2+\frac{f_1}{r_{21}}+\frac{f_3}{r_{23}}\right) \\ +f_3\left(\frac{f_1}{r_{13}r_{21}}+\frac{f_2}{r_{23}r_{12}}+\frac{f_3}{r_{13}r_{23}}\right)\left(f_3+\frac{f_1}{r_{31}}+\frac{f_2}{r_{32}}\right) \end{array}\right)} = 0 \tag{10}$$

$$F_3 - \frac{f_3\left(\frac{f_1}{r_{13}r_{21}}+\frac{f_2}{r_{23}r_{12}}+\frac{f_3}{r_{13}r_{23}}\right)\left(f_3+\frac{f_1}{r_{31}}+\frac{f_2}{r_{32}}\right)}{\left(\begin{array}{c} f_1\left(\frac{f_1}{r_{21}r_{31}}+\frac{f_2}{r_{21}r_{32}}+\frac{f_3}{r_{31}r_{23}}\right)\left(f_1+\frac{f_2}{r_{12}}+\frac{f_3}{r_{13}}\right) \\ +f_2\left(\frac{f_1}{r_{12}r_{31}}+\frac{f_2}{r_{12}r_{32}}+\frac{f_3}{r_{13}r_{32}}\right)\left(f_2+\frac{f_1}{r_{21}}+\frac{f_3}{r_{23}}\right) \\ +f_3\left(\frac{f_1}{r_{13}r_{21}}+\frac{f_2}{r_{23}r_{12}}+\frac{f_3}{r_{13}r_{23}}\right)\left(f_3+\frac{f_1}{r_{31}}+\frac{f_2}{r_{32}}\right) \end{array}\right)} = 0 \tag{11}$$

The cumulative terpolymer composition ($\overline{F_i}$) is defined via Equations 12–14, using the initial feed monomer mole fractions ($f_{i,0}$), the mole fraction of unreacted monomer within the polymerizing mixture ($f_i$), and the total molar conversion ($X_n$).

$$\overline{F_1} = \frac{f_{1,0}-f_1(1-X_n)}{X_n} \tag{12}$$

$$\overline{F_2} = \frac{f_{2,0}-f_2(1-X_n)}{X_n} \tag{13}$$

$$\overline{F_3} = \frac{f_{3,0}-f_3(1-X_n)}{X_n} \tag{14}$$

In addition, when a constant composition cannot be assumed, particularly when composition drift becomes significant, $f_i$ needs to be assessed with respect to the conversion trajectory, as depicted in Eqs. (15)– (17).

$$\frac{df_1}{dX_n} = \frac{f_1 - F_1}{1 - X_n} \tag{15}$$

$$\frac{df_2}{dX_n} = \frac{f_2 - F_2}{1 - X_n} \tag{16}$$

$$\frac{df_3}{dX_n} = \frac{f_3 - F_3}{1 - X_n} \tag{17}$$

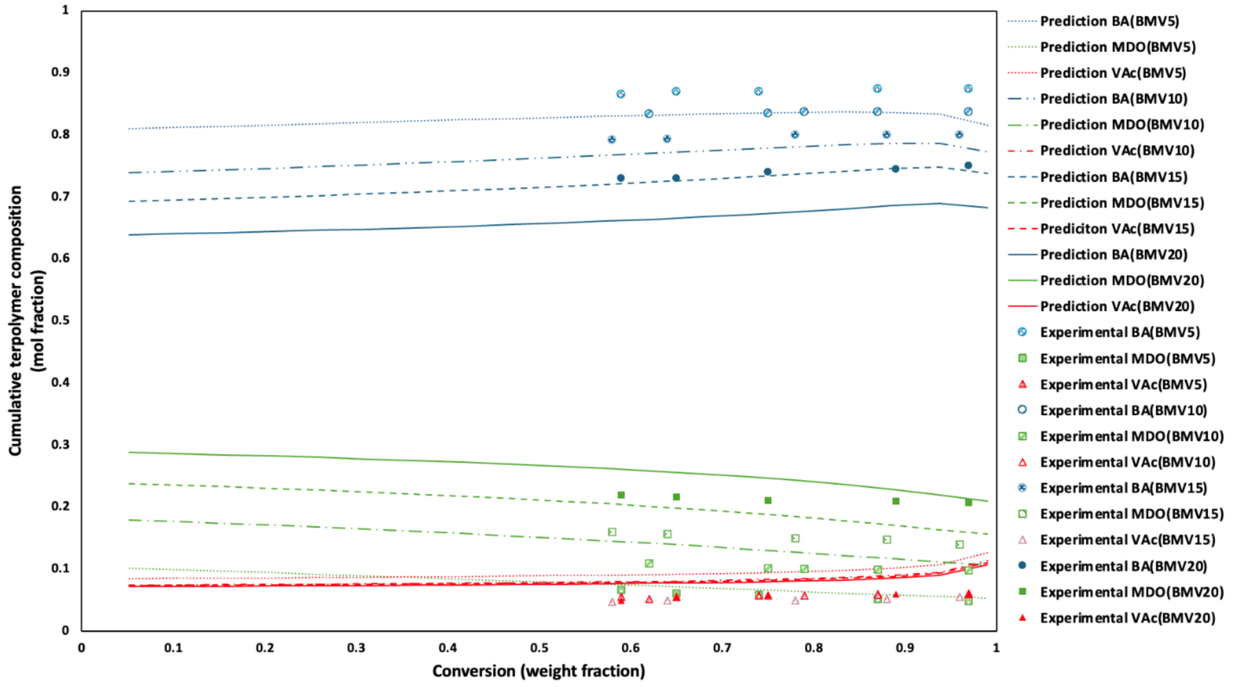


Figure S9. Model predictions and experimental data of cumulative terpolymer composition vs. conversion using ternary reactivity ratios for all terpolymers (BMV5, BMV10, BMV15, and BMV20).

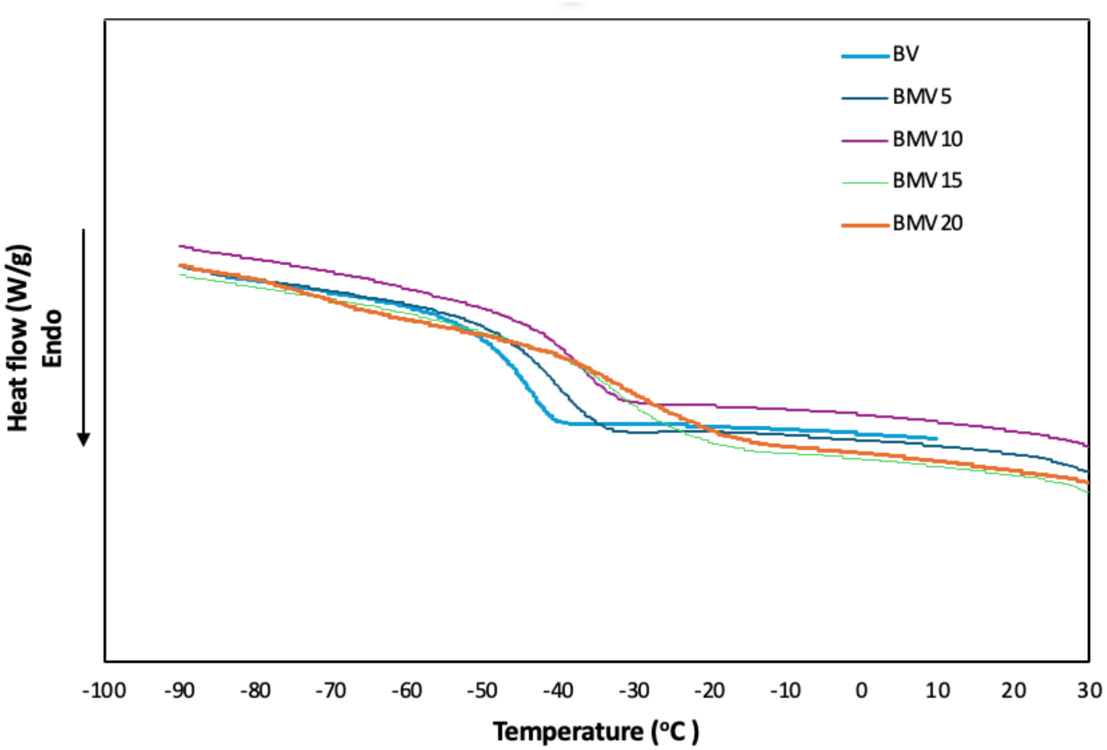


Figure S10. DSC thermograms of BV, BMV5, BMV10, BMV15, and BMV20 show each $T_g$ with a single transition peak.

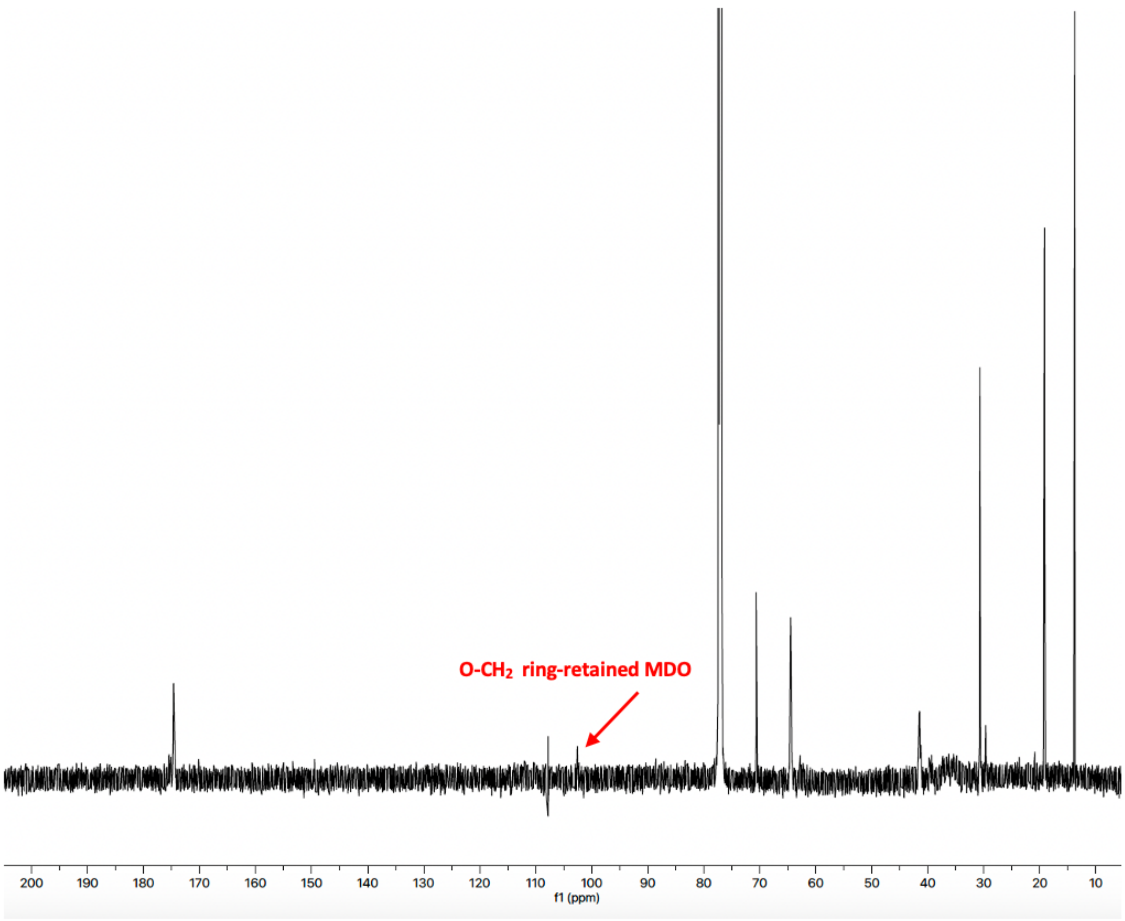


Figure S11. $^{13}$C-NMR spectrum for BMV5 in $CDCl_3$. The peak at 107 ppm is a narrow artifact observed in our $^{13}$C-NMR results using the AVIII600 NMR instrument.

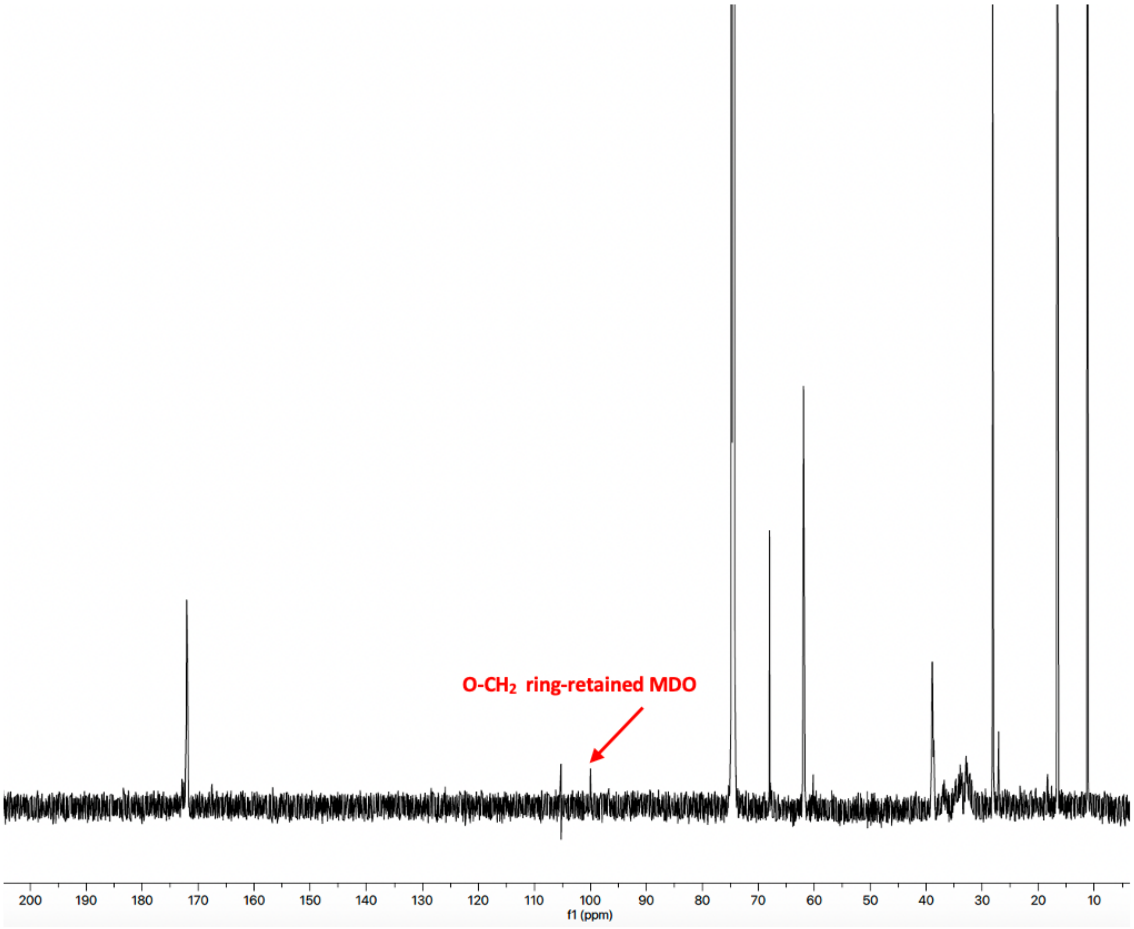


Figure S12. $^{13}$C-NMR spectrum for BMV10 in $CDCl_3$. The peak at 105 ppm is a narrow artifact observed in our $^{13}$C-NMR results using the AVIII600 NMR instrument.

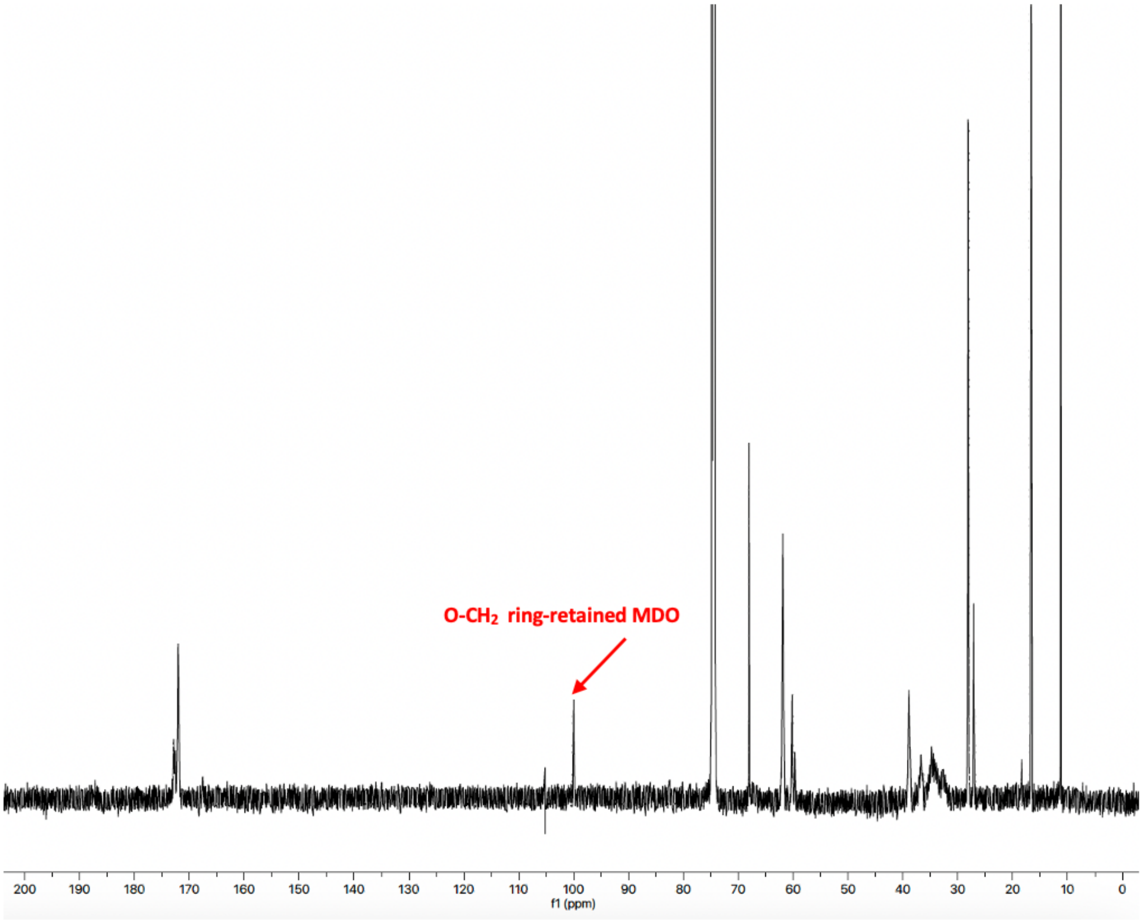


Figure S13. $^{13}$C-NMR spectrum for BMV15 in $CDCl_3$. The peak at 105 ppm is a narrow artifact observed in our $^{13}$C-NMR results using the AVIII600 NMR instrument.

The MDO ring-retention using $^{13}$C-NMR spectroscopy was calculated using four peaks shown in Figure 5 in the main manuscript. The peak at 100 ppm represents ring-retained MDO. By integrating each peak that represents the selected carbon atoms in the figure:

$$A_1 = n \quad (18)$$

$$A_2 = h \quad (19)$$

$$A_3 = k \quad (20)$$

$$A_4 = j \quad (21)$$

$$F_{\text{ring-retained MDO}} = n / (n + h + k + j) \tag{22}$$

where $n$, $h$, $k$ and $j$ relate to the ring-retained MDO, the ring-opened MDO, BA, and VAc carbons, respectively, found in areas $A_1$, $A_2$, $A_3$ and $A_4$. Area $A_1$ represents the carbon of ring-retained MDO ("d" in Figure 5 in the main manuscript), $A_2$ represents the ring-opened MDO ("b" in Figure 5), area $A_3$ represents the carbon of BA ("a" in Figure 5 in the main manuscript) and area $A_4$ represents the carbon of VAc ("e" in Figure 5 in the main manuscript). After solving for $n$, $h$, $k$ and $j$ (Equations 18-21), ring-retained MDO was calculated using Equation 22.

a)

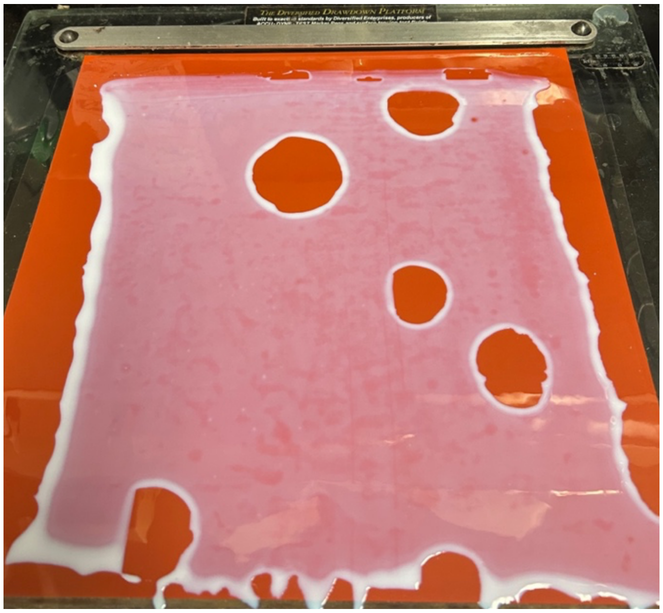

b)

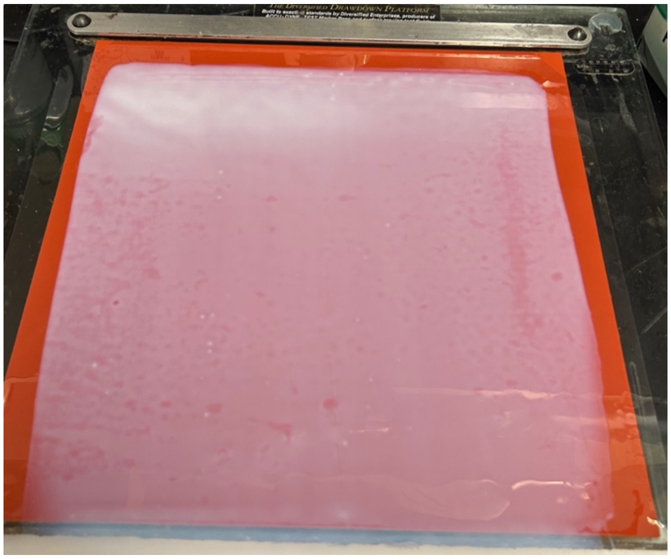

Figure S14. Cast films of BMV20 a) before and b) after adding wetting agent.

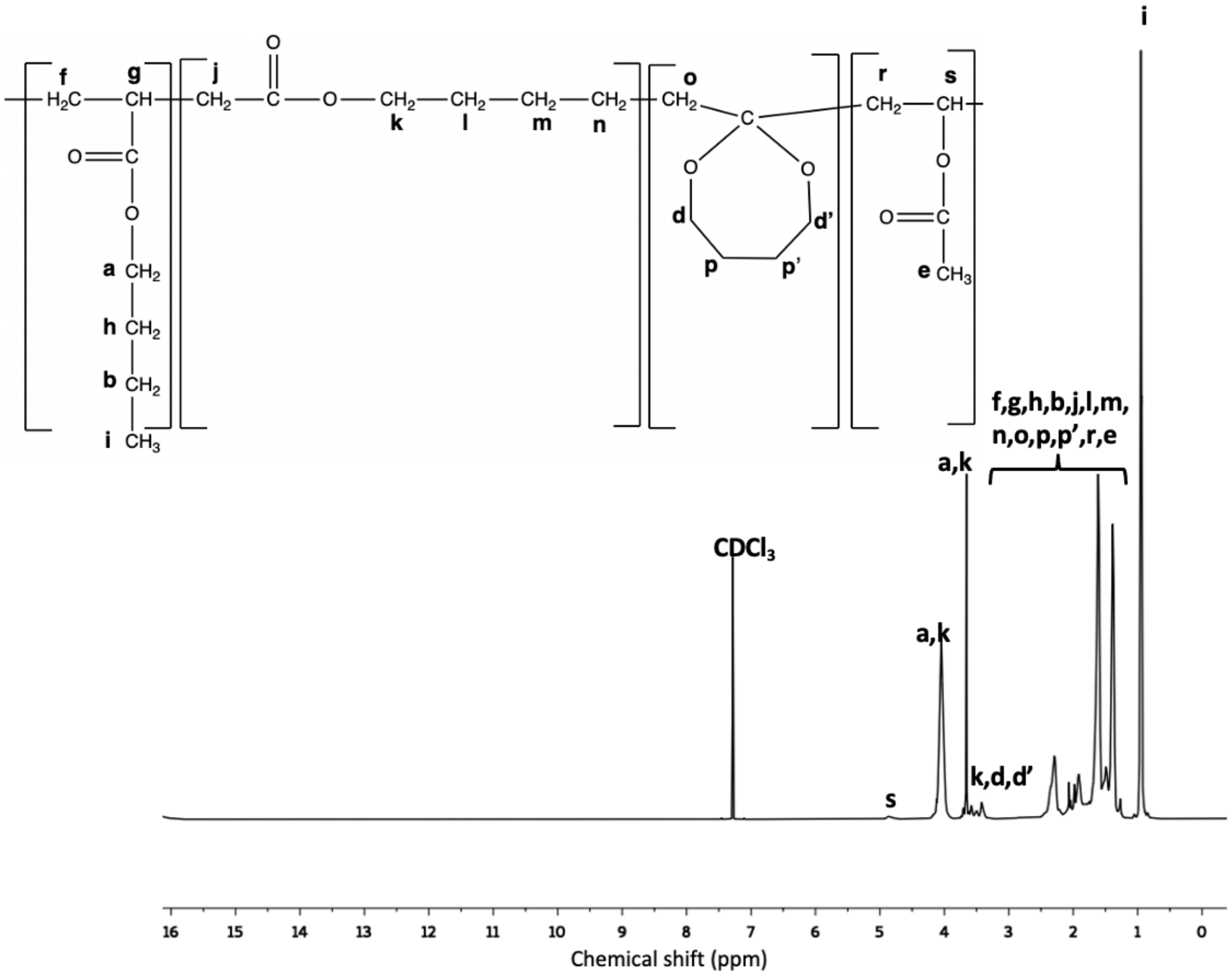


Figure S15. $^{1}$H-NMR spectrum for BMV10-NEW in $CDCl_3$. See Figure S5 for detailed peak assignments.

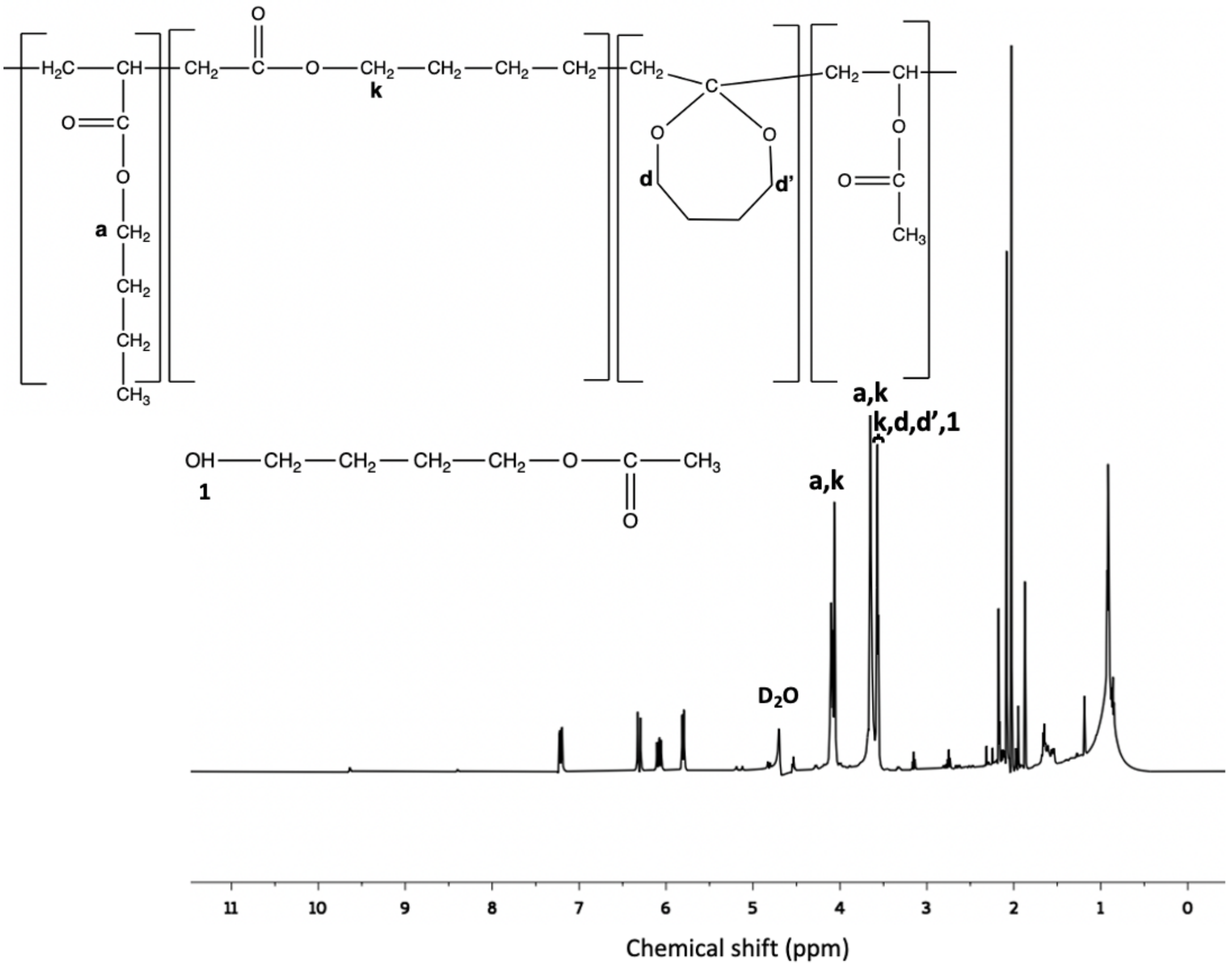


Figure S16. $^1$H-NMR Watergate spectrum of BMV10-NEW latex in $D_2O$.

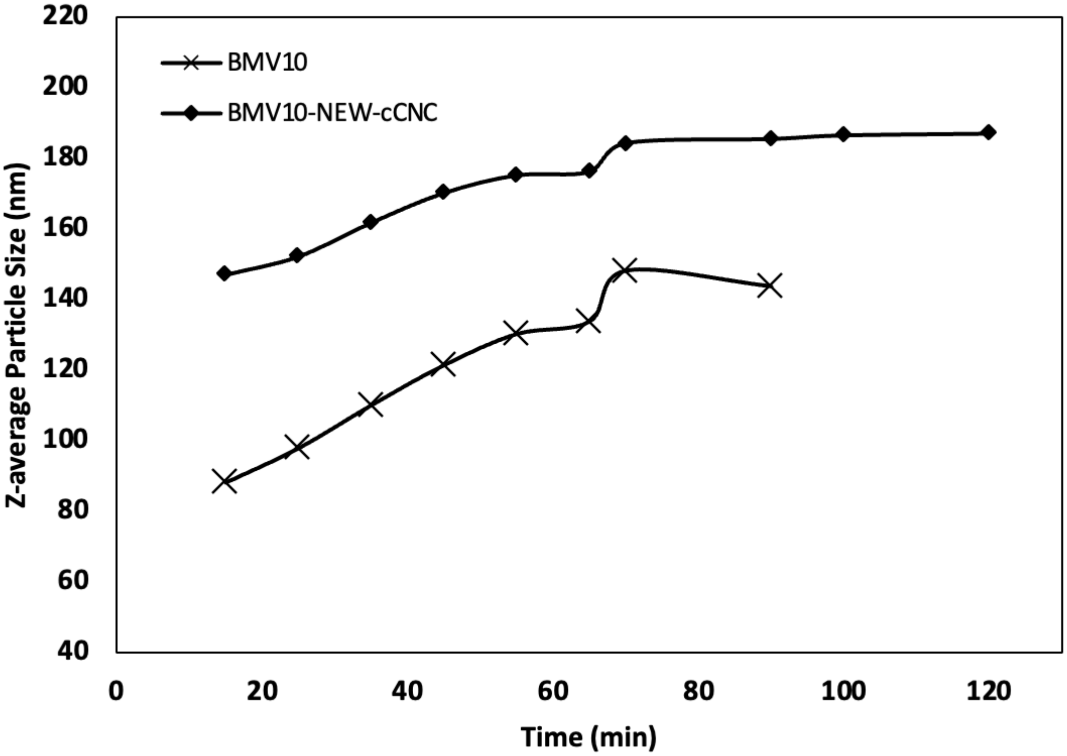


Figure S17. Z-average particle size versus time for BMV10 and BMV10-NEW

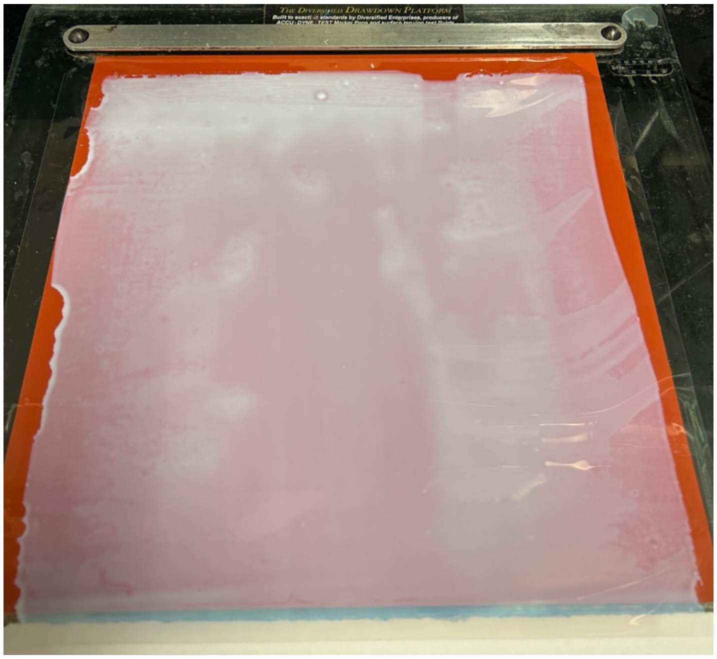

Figure S18. Cast film of BMV10-NEW-cCNC. No wetting agent was added.
